# RAB6 GTPase regulates mammary secretory function by controlling the activation of STAT5

**DOI:** 10.1242/dev.190744

**Published:** 2020-10-08

**Authors:** Surya Cayre, Marisa M. Faraldo, Sabine Bardin, Stéphanie Miserey-Lenkei, Marie-Ange Deugnier, Bruno Goud

**Affiliations:** 1 Department of Cell Biology and Cancer, Institut Curie, PSL Research University, Sorbonne Université, CNRS, UMR144, Paris F-75005, France; 2 INSERM, Paris F-75013, France

**Keywords:** RAB6 GTPase, Mammary gland, Luminal secretory cells, STAT5, PRL signaling

## Abstract

The Golgi-associated RAB GTPases, RAB6A and RAB6A′, regulate anterograde and retrograde transport pathways from and to the Golgi. *In vitro*, RAB6A/A′ control several cellular functions including cell division, migration, adhesion and polarity. However, their role remains poorly described *in vivo*. Here, we generated BlgCre; *Rab6a*^F/F^ mice presenting a specific deletion of *Rab6a* in the mammary luminal secretory lineage during gestation and lactation. *Rab6a* loss severely impaired the differentiation, maturation and maintenance of the secretory tissue, compromising lactation. The mutant epithelium displayed a decreased activation of STAT5, a key regulator of the lactogenic process primarily governed by prolactin. Data obtained with a mammary epithelial cell line suggested that defective STAT5 activation might originate from a perturbed transport of the prolactin receptor, altering its membrane expression and signaling cascade. Despite the major functional defects observed upon *Rab6a* deletion, the polarized organization of the mammary epithelial bilayer was preserved. Altogether, our data reveal a crucial role for RAB6A/A′ in the lactogenic function of the mammary gland and suggest that the trafficking pathways controlled by RAB6A/A′ depend on cell-type specialization and tissue context.

## Abstract (precis)

**Summary:** This study reveals a role for the Golgi-associated RAB GTPases RAB6A/A′ in the lactogenic function of the mammary gland.

## INTRODUCTION

Small GTPases of the RAB family are master regulators of vesicular transport and membrane trafficking in eukaryotic cells. Through their specific intracellular localization and ability to recruit various types of effectors, RAB GTPases tightly control the molecular exchanges between cell compartments (Stenmark, 2009). In human, this large family comprises about 70 members which include four RAB6 proteins, RAB6A and its splice variant RAB6A′, RAB6B and RAB6C. The ubiquitously expressed RAB6A/A′ are the most abundant Golgi-associated RAB GTPases and have been reported to control anterograde and retrograde trafficking from and to the Golgi apparatus (Goud et al., 2018).

RAB6A/A′ have been intensively studied in cultured cells and implicated in several cellular functions, including cell division, migration, adhesion, polarity establishment and secretory mechanisms (Miserey-Lenkei et al., 2006, 2010; Micaroni et al., 2013; Shafaq-Zadah et al., 2016; Fourriere et al., 2019; Homma et al., 2019). Importantly, we recently reported that RAB6A/A′ are essential for early embryonic development. RAB6A/A′ null mouse embryos die at day 5.5, exhibiting defective adhesion between the epiblast layer and the visceral endoderm, with a disorganization of the basement membrane and a perturbed expression of the β1 integrin chain in epiblast cells (Shafaq-Zadah et al., 2016).

RAB6 function in highly polarized epithelial tissues still remains poorly documented. To investigate the role of RAB6A/A′ in a tissue-specific context, we have generated *Rab6a*^F/F^ mice carrying a conditional null allele of *Rab6a* that allows the targeted deletion of RAB6A/A′ (Bardin et al., 2015). Using this model, we have shown that RAB6A/A′ is required for the proper maturation of melanosomes in melanocytes and that they play a key role in T lymphocyte activation (Patwardhan et al., 2017; Carpier et al., 2018).

The mammary gland provides a unique model to study morphogenesis, epithelial tissue polarity, cell fate specification and secretory mechanisms. Its development is mainly postnatal and comprises two distinct morphogenetic events: the growth and branching of epithelial ducts during puberty and the lobulo-alveolar development during gestation (Macias and Hinck, 2012; Brisken and Ataca, 2015). At each pregnancy, under progesterone and prolactin (PRL) stimulation, the mammary epithelium undergoes proliferation and differentiation, preparing it for milk secretion, its primary function. At parturition, a fall in progesterone accompanied by elevated PRL levels triggers secretory activation and lactation (Anderson et al., 2007). PRL and its receptor (PRLR) play a pivotal role in alveologenesis and lactation, in particular through activation of the transcription factor STAT5 (Hennighausen and Robinson, 2008).

In ducts and alveoli, the mammary epithelium comprises an outer layer of basal myoepithelial cells and an inner layer of luminal cells organized around a lumen. This bilayered structure sits on a basement membrane surrounded by stromal elements. During lactation, the secretory luminal cells produce milk components, including milk proteins, lactose and lipids, whereas the contractile myoepithelial cells serve for milk expulsion (Anderson et al., 2007; Moumen et al., 2011). Luminal cells are highly polarized, with an apical domain facing the lumen and a basolateral domain contacting adjacent luminal and myoepithelial cells. In addition, during lactation secretory luminal cells interact with the basement membrane myoepithelial cells forming a discontinuous layer around alveoli. Owing to their specialized function and numerous interactions, luminal cells display a large set of apical and baso-lateral surface proteins (Glukhova and Streuli, 2013; Chatterjee and McCaffrey, 2014).

The mammary luminal cell population is heterogeneous, comprising hormone-sensing cells positive for estrogen and progesterone receptors (ER, PR) and cells devoid of ER/PR expression (Brisken and Ataca, 2015). The secretory lineage is largely composed of ER^−^/PR^−^ cells. It contains distinct stem/progenitor cells that are amplified during gestation and give rise to fully functional secretory cells in the lactating gland (Rodilla et al., 2015; Bach et al., 2017). To study the role of RAB6A/A′ in mammary gland development and function, we generated BlgCre; *Rab6a*^F/F^ mice, in which *Rab6a* was deleted specifically in the secretory lineage via the β-lactoglobulin (*Blg*) promoter, primarily active throughout pregnancy and lactation (Selbert et al., 1998; Chapman et al., 1999; Naylor et al., 2005; Molyneux et al., 2010; Romagnoli et al., 2020).

## RESULTS

### *Rab6a* expression is upregulated in the luminal secretory lineage during gestation

**Fig. 1.**
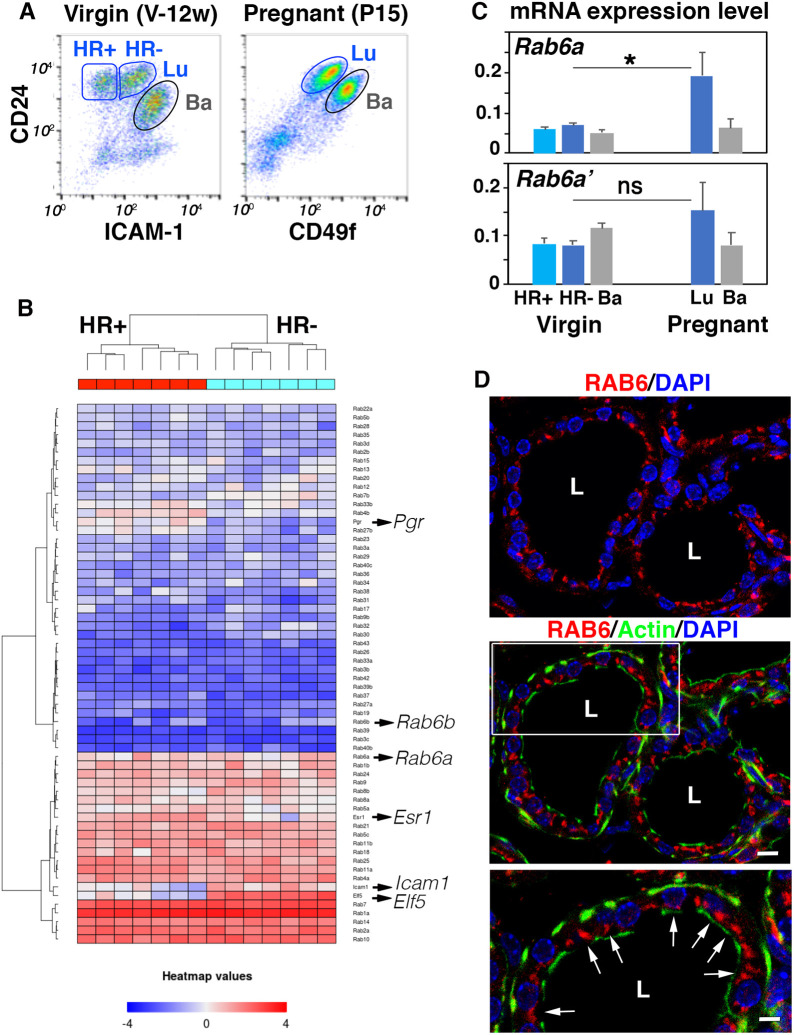
***Rab6* expression in mammary luminal and basal cells isolated from adult virgin and pregnant females.** (A) Flow cytometry analysis of mammary cells isolated from 12-week-old virgin mice (left; V-12w) and 15-day pregnant mice (right, P15). The gated subsets within the CD24^+^ epithelial cell pool are: CD24^low^ ICAM1^+^ basal cells (Ba), CD24^high^ ICAM1^−^ and CD24^high^ ICAM1^+^ luminal cells (Lu; HR^+^ and HR^−^, respectively) (left); CD24^low^ CD49f^high^ basal cells (Ba) and CD24^high^ CD49f^low^ (Lu) (right). (B) Heat map based on microarray data (Chiche et al., 2019) showing the relative gene expression of 58 RAB GTPases in the HR^+^ and HR^−^ luminal cell populations isolated from adult virgin mice as shown in A (left). Genes expressed at a significant level appear in pink to red. The heat map includes the reference genes *Icam1*, *Elf5*, *Pgr* (encoding PR) and *Esr1* (encoding ER) used to characterize the luminal cell populations. *Pgr* and *Esr1* are highly expressed in the hormone-responsive HR^+^ cells, whereas *Elf5* indicates the HR^−^ luminal secretory lineage. Microarray data are from seven separate sorting experiments. Each cell preparation was obtained from a pool of mammary glands taken from 3-4 females. (C) qPCR analysis of *Rab6a* and *Rab6a*′ expression in mammary basal and luminal cells isolated from adult virgin and pregnant mice as shown in A. Data are mean±s.e.m. of three separate cell preparations. For the virgin state, each preparation was obtained from a pool of mammary glands taken from 3-4 adult virgin females (total number=10). At pregnancy, each preparation was obtained from a pool of mammary glands taken from one female (total number=3). *P=0.039. ns, not significant. (D) RAB6 immunolocalization in a lactating mammary gland. Actin marks basal myoepithelial cells and the apical pole of luminal cells facing the alveolar lumen (L). Nuclei are stained with DAPI. Some of them are out of the focal plane. The lowest panel shows an enlarged view of the boxed area in the middle panel. Arrows point to the apical presence of RAB6. Scale bars: 10 µm in D (top and middle); 5.5 µm in D (bottom).

To get insight into RAB6 GTPase expression in mammary luminal cells, we first analyzed their transcriptomic profiles. In the adult virgin gland, the ER^−^/PR^−^ luminal cell population, targeted by the *Blg* promoter, can be separated from the ER^+^/PR^+^ cell fraction using flow cytometry. As previously described (Di-Cicco et al., 2015), we used ICAM1 expression combined with the epithelial-specific marker CD24 to discriminate CD24^low^ ICAM1^+^ basal cells, CD24^high^ ICAM1^−^ luminal cells (ER^+^/PR^+^, referred to as HR^+^) and CD24^high^ ICAM1^+^ luminal cells (mostly ER^−^/PR^−^; referred to as HR^−^) (Fig. 1A). Using the transcriptomic profiles of the HR^+^ (ICAM1^−^) and HR^−^ (ICAM1^+^) luminal cell subsets that we previously established (Chiche et al., 2019), we analyzed the expression levels of the 58 Rab GTPase genes present in the microarrays. Only 19 Rab genes were found robustly expressed in both HR^+^ and HR^−^ luminal cell populations (Fig. 1B and Table S1). In addition to *Rab6a*, these include *Rab1a/b*, *Rab2a*, *Rab4a*, *Rab5a/c*, *Rab7a* (*Rab7*), *Rab8a/b*, *Rab9a* (*Rab9*), *Rab10*, *Rab11a/b*, *Rab14*, *Rab18*, *Rab21*, *Rab24* and *Rab25*. HR^+^ and HR^−^ luminal cell populations displayed very similar *Rab* profiles, with *Rab1a*, *Rab2a*, *Rab7a*, *Rab10* and *Rab14* being the top ranked genes. Of note, they did not significantly express *Rab6b* (Fig. 1B and Table S1), known to be mainly expressed in neuronal and neuro-endocrine cells (Opdam et al., 2000; Goud et al., 2018).

*Rab6a* encodes two Golgi-associated isoforms, RAB6A and RAB6A′. They only differ by three amino acids and are both ubiquitously expressed (Goud et al., 2018). To complement the microarray analysis, we compared the expression levels of *Rab6a* and *Rab6a*′ transcripts in the mammary cell populations isolated from virgin glands, using qPCR. Similar amounts of *Rab6a* and *Rab6a*′ transcripts were detected in HR^−^ as in HR^+^ luminal cells (Fig. 1C). The two variants were expressed in basal cells at levels close to luminal cells (Fig. 1C).

To further investigate *Rab6a* expression in the mammary epithelium, we isolated luminal and basal cells from mice at day 15 of pregnancy (P15) (Fig. 1A), using differential expression of CD24 and α6 integrin chain (CD49f; ITGA6) as described previously (Visvader and Stingl, 2014). At this stage, the luminal population is largely composed of HR^−^ cells that have been amplified upon hormonal stimulation and are committed to the secretory lineage (Macias and Hinck, 2012). Interestingly, qPCR data showed that luminal cells from pregnant mice displayed higher levels of *Rab6a* than those from adult virgin mice (Fig. 1C). *Rab6a*′ expression in luminal cells exhibited the same tendency as that of *Rab6a* (Fig. 1C). *Rab6a* and *Rab6a*′ levels were not significantly changed in basal cells upon pregnancy (Fig. 1C).

Overall, these data demonstrate that *Rab6a/a*′ expression is upregulated during pregnancy in luminal cells only and therefore suggest a role for RAB6A/A′ in the secretory lineage. Consistently, using an antibody recognizing all RAB6 isoforms, we observed a strong RAB6 expression in the luminal secretory cells of the lactating gland (Fig. 1D). As expected for a Golgi-associated protein, RAB6 was concentrated at the apical pole of the luminal cells.

### Mammary development and epithelium organization of BlgCre; *Rab6a*^F/F^ mice are not critically affected at P15

*Blg* promoter activity has been reported to be weak in the mammary tissue of virgin females, with a substantial increase from mid-pregnancy (∼P10) and peak during lactation (Selbert et al., 1998; Chapman et al., 1999; Naylor et al., 2005; Romagnoli et al., 2020). To evaluate the role of RAB6A/A′ (hereafter referred to as RAB6A) in the development of the luminal secretory lineage, we first analyzed the mammary phenotype of BlgCre; *Rab6a*^F/F^ mutant mice at P15, using *Rab6a*^F/F^ littermates as controls. Quantitative flow cytometry analyses showed that mutant and control mammary tissues displayed similar percentages of CD24+ epithelial cells with identical luminal/basal cell ratios (Fig. S1A,B). Surface expression of α6 or β1 integrin chains was not perturbed in mutant luminal and basal cells (Fig. S1A). The extent of *Rab6a* deletion, checked in mutant luminal cells by qPCR, was estimated at 80% (Fig. S1C), in agreement with previous studies using BlgCre-mediated gene deletion (Chapman et al., 1999; Naylor et al., 2005; Romagnoli et al., 2020).

In line with the flow cytometry data, whole-mount analyses did not reveal differences between mutant and control mammary trees that displayed similar fat pad occupancy and branching patterns (Fig. S1D). Organization of the mammary bilayer, evidenced by double labeling for the luminal- and basal-specific keratins K8 and K5, was not perturbed in mutant mice (Fig. S1D). As expected at this developmental stage, mutant and control mammary epithelium were actively proliferating (Fig. S1D). They both displayed typical alveolar buds with nascent cytoplasmic lipid droplets (CLD) positive for adipophilin (ADPH; PLIN2) (Fig. S1E), a major CLD protein, the expression of which is specifically induced in alveolar luminal cells around mid-pregnancy (Anderson et al., 2007; Russell et al., 2011). Percentages of PR^+^ luminal cells were similar in mutant and control epithelium (Fig. S1F), indicating that the relative proportion of HR^−^ and HR^+^ luminal lineages was not affected upon *Rab6a* deletion.

Collectively, these data indicated that *Rab6a* deletion in luminal cells did not critically impact the first set of pregnancy-associated developmental events, resulting in amplification of basal and luminal cell populations, side branching and alveolar bud formation. Moreover, the bilayered organization of the mammary epithelium and expression of luminal cell markers, such as K8, ADPH, α6 and β1 integrin chains, were preserved upon *Rab6a* deletion.

### BlgCre; *Rab6a*^F/F^ mice exhibit altered alveolar differentiation at late gestation

We next analyzed the mammary phenotype of BlgCre; *Rab6a*^F/F^ mutant mice at P18. This prelactational stage is characterized by well described histological changes and secretory differentiation events including alveolar distension with luminal space enlargement, presence of large CLDs in alveolar luminal cells and substantial milk protein expression, in particular caseins, whey acidic protein (WAP) and α-lactalbumin, an essential cofactor for milk lactose synthesis (Oakes et al., 2006, 2017; Anderson et al., 2007).

**Fig. 2.**
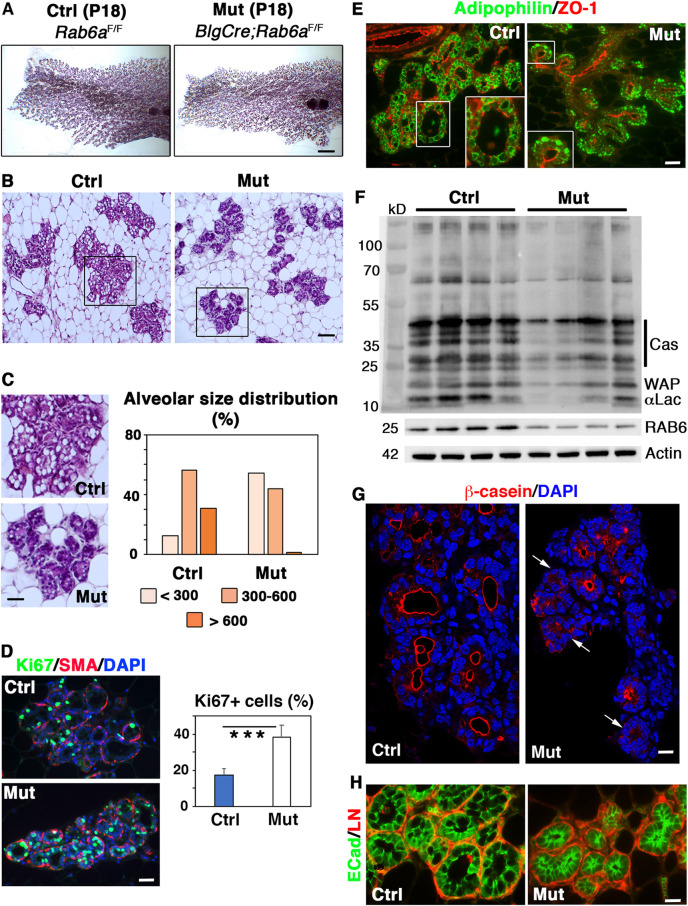
**Impaired alveolar luminal cell differentiation in Blg-Cre; *Rab6a*^F/F^ females at late pregnancy (P18).** (A) Carmine-stained wholemounts of mammary glands from control and mutant females at P18. (B) Views of H&E-stained histological sections through control and mutant mammary glands at P18. (C) Enlarged views of control and mutant alveoli shown in B (left) and compared distributions of alveolar size at P18, estimated in µm (right). Data are from a pool of measurements performed on two distinct histological sections from three control and three mutant mammary glands. A total of 1450 alveoli was measured. Pearson's Chi-square test: *P*<0.001. (D) Cell proliferation in control and mutant mammary glands at P18, estimated by Ki67 expression. Triple Ki67/SMA/DAPI immunofluorescent staining (left). SMA marks basal myoepithelial cells surrounding alveoli. Percentages of Ki67+ luminal cells (right). Data are mean±s.e.m. of counts performed on sections through three distinct control and mutant mammary glands. Around 2000 DAPI-stained nuclei were counted on each section. ***P<0.0001. (E) Immunolocalization of adipophilin and ZO-1 in control and mutant mammary tissues at P18, with insets showing enlarged views of individual alveoli. (F) Western blots for milk proteins, RAB6 and actin (used as loading control) performed with four distinct control and mutant mammary gland protein extracts. Molecular weight (MW) markers are shown on the left and the corresponding proteins, α-lactalbumin (αLac), whey acidic protein (WAP), caseins (Cas) on the right. MW of RAB6 and actin are indicated. Quantification of RAB6 depletion is shown in Fig. S2A. (G) Immunolocalization of β-casein in control and mutant mammary epithelium at P18. Arrows indicate the mutant alveoli devoid of β-casein expression. (H) Double immunolabeling of E-cadherin and laminin (ECad/LN) in control and mutant mammary tissues at P18. Scale bars: 2.25 mm in A; 100 µm in B; 48 µm in C; 35 µm in D; 45 µm in E; 20 µm in G; 15 µm in H.

Whole-mount images indicated that the mutant epithelium, although well developed, appeared less dense than the control (Fig. 2A). Quantification of Hematoxylin & Eosin (H&E)-stained tissue sections showed a significant reduction in mammary fat pad occupancy by the mutant epithelium (Fig. S2A). Notably, alveoli from mutant glands were condensed compared with those from control glands (Fig. 2B), evoking reduced amounts of secretory products. Analysis of alveolar size distribution confirmed that, unlike controls, the mutant glands contained a majority of alveoli of small to medium size and almost no large alveoli (Fig. 2C). This phenotype was not associated with reduced proliferation capacities as the proliferation index was twice as high in the mutant compared with the control epithelium (Fig. 2D), indicating that RAB6A-deficient mammary glands, unlike controls, were still in an active growing phase.

Notably, H&E staining revealed that alveoli from the mutant tissue displayed a low number of CLDs (Fig. 2C). Immunofluorescent studies confirmed the presence of large ADPH-coated CLDs in alveolar luminal cells from control glands (Fig. 2E). By contrast, mutant alveolar luminal cells primarily contained small ADPH-expressing CLDs often located at the basal pole (Fig. 2E). Thus, RAB6A deficiency appeared to impair or considerably delay CLD maturation, indicating perturbed secretory differentiation events.

To further analyze the differentiation features characterizing late gestation, we performed western blots (WB) on mammary gland extracts using an antibody against mouse milk-specific proteins, as previously reported (Oakes et al., 2017). The data showed that the mutant samples contained lower amounts of the milk components α-lactalbumin, caseins and WAP than the controls (Fig. 2F). Efficiency of RAB6 depletion was controlled in parallel using an antibody recognizing RAB6A/A′ and RAB6B (Fig. 2F and Fig. S2B). In line with the WB data, immunolocalization studies showed that, in the mutant tissue, numerous alveoli lacked β-casein expression, whereas in controls almost all alveoli displayed a strong β-casein staining lining the lumen (Fig. 2G).

Immunodetection of lineage-specific markers showed that, overall, the polarized organization of the mutant mammary epithelium was preserved at late pregnancy. In mutant as in control alveoli, luminal cells (K8^+^, E-cadherin^+^) were surrounded by basally-located myoepithelial cells expressing K5 and smooth-muscle actin (SMA) (Fig. S2C). Laminin, a major component of the basement membrane, appeared normally deposited around the alveoli of the mutant tissue (Fig. 2H). Major markers of apical and baso-lateral polarity, such as ZO-1 (TJP1), MUC1, E-cadherin and β1 integrin, were strongly expressed and properly localized in the mutant alveolar luminal cells (Fig. 2H and Fig. S2C-E). ZO-1 and MUC1 staining well illustrated the reduced luminal space of the condensed mutant alveoli (Fig. S2D). Flow cytometry analyses confirmed that surface expression of α6 and β1 integrin chains was not perturbed in the mutant epithelium, neither in luminal nor in basal cells (Fig. S2F).

Together, the data obtained at late pregnancy revealed impaired or delayed differentiation events in BlgCre; *Rab6a*^F/F^ mammary glands. In particular, the mutant epithelium exhibited deficient CLD maturation and decreased milk protein production, most likely accounting for the defective alveolar enlargement. This indicated an important role for RAB6A in the alveolar differentiation stage characterizing the prelactational phase. Noticeably, perturbations induced by loss of *Rab6a* occurred without any obvious alterations of the bilayered organization of the mammary epithelium and the apico-basal polarity of the alveolar luminal cells, suggesting functional rather than structural defects.

### BlgCre; *Rab6a*^F/F^ mice display a severe lactation defect

**Fig. 3.**
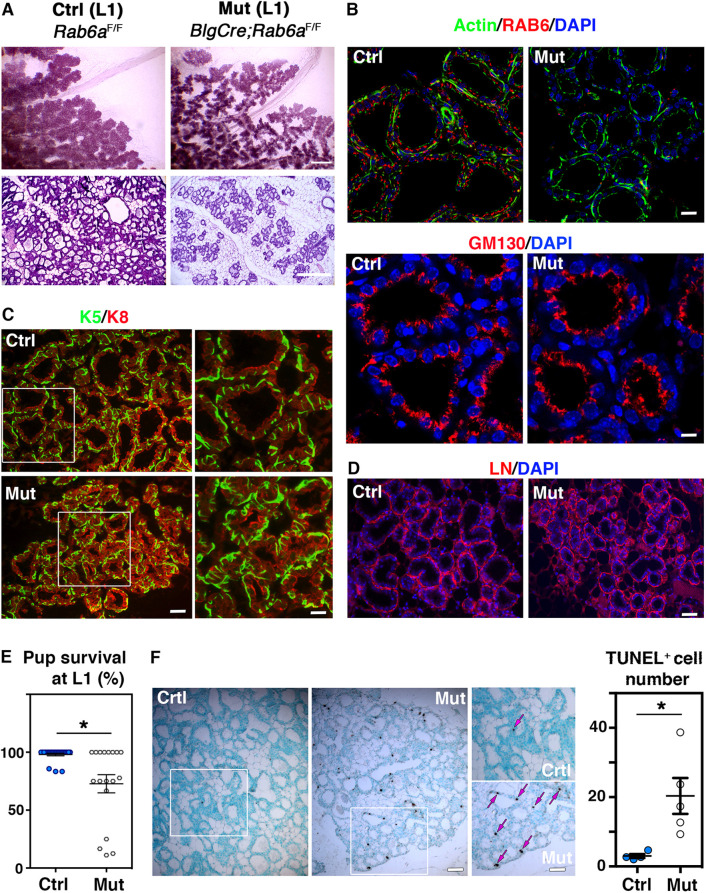
**Mammary phenotype and lactation deficiency of Blg-Cre; *Rab6a*^F/F^ females at L1.** (A) Carmine-stained wholemounts (upper panels) and H&E-stained histological sections (lower panels) of mammary glands from control and mutant females at L1. (B) RAB6 and GM130 immunodetection in control and mutant alveolar luminal cells at L1. Upper panels: triple actin/RAB6/DAPI staining. Actin marks basal myoepithelial cells and the apical membrane of luminal cells. RAB6 is absent from mutant luminal cells but detected in basal myoepithelial cells. Lower panels: double GM130/DAPI staining showing similar apical Golgi distribution in control and mutant luminal cells. (C) Immunolabeling of basal-specific (K5) and luminal-specific (K8) keratins in control and mutant mammary epithelium at L1. Right panels show enlarged views of the boxed areas in left panels. Basal myoepithelial cells display a typical stellate shape in both control and mutant samples. (D) Immunodetection of laminin (LN) in control and mutant alveoli at L1 showing similar deposition. (E) Survival of pups nursed by control and mutant primiparous dams at L1. Litters from 24 control and 18 mutant females were analyzed. Each point of the graph represents one dam. The percentages reflect the proportion of healthy, viable pups within each individual litter. The dams nursing their entire litter are plotted at 100%, those unable to feed all their pups below 100%. *P=0.05. (F) Cell death in control and mutant mammary epithelium at L1, as revealed by TUNEL assay. Left panels show views of sections through control and mutant glands. Methyl green was used as counterstaining. Enlarged views of the boxed areas in left panels are shown in the right panels. Arrows indicate TUNEL-positive cells. Quantification shows number of TUNEL-positive cells per low-magnification microscopic field. Data are mean±s.e.m. from four control and five mutant mice. Three distinct fields per mouse were quantified. *P=0.03. Scale bars: 0.3 mm in A (upper); 300 µm in A (lower); 20 µm in B (upper); 12 µm in B (lower); 40 µm in C (left); 20 µm in C (right); 75 µm in D and F (left); 55 µm in F (right).

In primiparous control mice, at day one of lactation (L1), the lobulo-alveolar structures almost entirely occupied the mammary fat pad and the milk-producing alveoli were markedly dilated, as seen on whole-mounts and histological sections (Fig. 3A and Fig. S3A). By contrast, the mammary epithelium from BlgCre; *Rab6a*^F/F^ mutant mice showed a significant reduction in fat pad occupancy (Fig. 3A and Fig. S3B) and, as observed at late gestation, the mutant tissue was devoid of large alveoli (Fig. 3A and Fig. S3C). Immunohistofluorescence studies showed that, as expected, RAB6 was absent from the secretory luminal cells of BlgCre; *Rab6a*^F/F^ mice at L1, whereas it was detected in the non-targeted basal myoepithelial cells (Fig. 3B)*.* Alveolar luminal cells lacking RAB6 exhibited a normal distribution of GM130 (GOLGA2), a cis-Golgi marker preferentially localized at their apical pole (Fig. 3B)*.* The bilayered organization of the mutant mammary epithelium was conserved and although poorly distended, alveoli were decorated with myoepithelial cells displaying a stellate shape, typical of the lactation period (Fig. 3C)*.* Mutant and control alveoli were enveloped by a continuous basement membrane rich in laminin (Fig. 3D).

We next investigated the ability of BlgCre; *Rab6a*^F/F^ females to support growth of a first litter. The mean number of pups per litter was similar for the mutant and control dams analyzed (7.5±0.39 and 6.8±0.32, respectively; mean±s.e.m.). However, whereas almost all control dams fed complete litters of healthy pups, non-viable newborns lacking milk in their stomach were frequently observed within litters fed by mutant dams (Fig. 3E). Ability of the mutant females to nurse varied from one dam to another: out of the eighteen mutant dams analyzed at L1, eight supported their entire litters, six properly fed about 75% of their pups and four fed only 10-20% of their pups. Similar variations in phenotype expression have been observed in a mouse model with a BlgCre-driven loss of β1-integrin (Naylor et al., 2005). To obtain representative data, we focused our analyses on the prevailing group of mutant dams, i.e. that feeding 75-100% of pups at L1.

Beyond L1, ethical reasons led us to eliminate most of the pups nursed by mutant dams, their survival being compromised by malnourishment. At L6, only four out of the eleven (36.4%) mutant dams examined were still able to feed pups (Fig. S3D). Two of them were analyzed and although nursing three and five pups respectively, they exhibited a deeply altered lobuloalveolar development (Fig. S3E). Moreover, their pups weighed significantly less than those nursed by control mothers (Fig. S3F). The mutant dams did not exhibit any obvious abnormal maternal nurturing behavior.

As perturbed lobulo-alveolar development and lactation defects are known to trigger early cell death, we analyzed cell death rates in mutant and control mammary tissues at L1, using TUNEL assays. The data revealed that, unlike controls, the RAB6A-depleted tissue sections contained numerous single TUNEL-positive luminal cells distributed throughout the epithelium (Fig. 3F). The lumen space of the mutant alveoli did not contain shed cells, indicating a non-massive cell death process. Noticeably, despite an abnormal cell death, the mutant secretory tissue had a higher proliferation index than the control (Fig. S3G), as observed at P18.

Collectively, these data showed that loss of *Rab6a* in the luminal secretory lineage severely compromised lactation and induced early cell death in primiparous females as early as postpartum day 1.

### Secretory activation is impaired at the onset of lactation in BlgCre; *Rab6a*^F/F^ mice

**Fig. 4.**
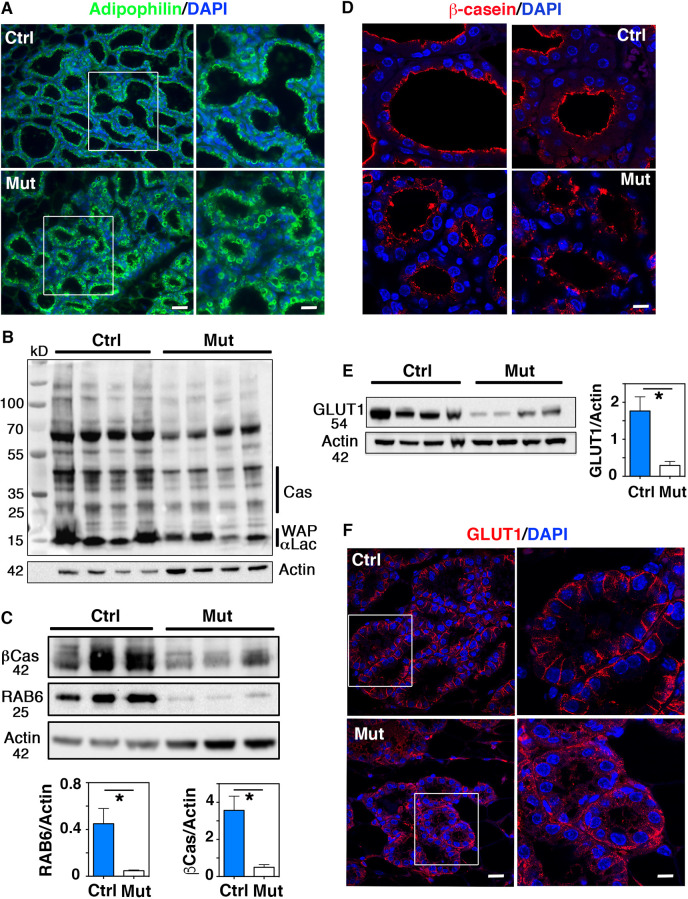
**Defective post-partum secretory activation of alveolar luminal cells in Blg-Cre; *Rab6a*^F/F^ females.** (A) Immunolocalization of adipophilin in control and mutant mammary epithelium at L1. Right panels show enlarged views of the boxed areas in left panels. Adipophilin-coated CLDs are retained in the cytosol of mutant alveolar luminal cells. (B) Western blots for milk proteins and actin (used as loading control) in four distinct control and mutant mammary gland extracts at L1. Molecular weight (MW) markers are shown on the left and the corresponding milk proteins on the right. (C) Western blots for β-casein (βCas), RAB6 and actin in three distinct control and mutant mammary gland extracts at L1. The lower panels show the related quantifications (mean±s.e.m.) normalized on actin amounts. *P=0.05 (RAB6), *P=0.026 (βCas). (D) Immunolocalization of β-casein in control and mutant mammary epithelium at L1. Two representative fields are shown. (E) Western blots for GLUT1 and actin in the four control and mutant mammary gland extracts analyzed in B. The histogram shows GLUT1 quantification (mean±s.e.m.) normalized on actin amounts. *P=0.026. (F) Immunolocalization of GLUT1 in control and mutant mammary epithelium at L1. Right panels show enlarged views of the boxed alveoli in left panels. In control, GLUT1 is sharply expressed at the baso-lateral membrane of alveolar luminal cells, whereas its expression is disorganized in mutant. MW are indicated in kD. Scale bars: 30 µm in A (left); 15 µm in A (right); 14 µm in D; 20 µm in F (left); 8 µm in F (right).

Transition from pregnancy to lactation is marked by changes in the size and cellular distribution of the luminal CLDs that are considered as indicators of proper secretory activation (Anderson et al., 2007). ADPH staining performed in control mammary glands at L1 showed that, as expected, the large CLDs visible at late pregnancy have been replaced by numerous smaller CLDs targeted to the apical surface of the luminal secretory cells (Fig. 4A). Unlike controls, mutant luminal cells displayed CLDs of various size, including large ones that accumulated in the cytosol (Fig. 4A). This abnormal CLD retention strongly suggests a failure in secretory activation in RAB6A-depleted alveolar luminal cells.

As observed at late gestation, WB analysis at L1 showed that, compared with control, the mutant mammary tissue contained lower amounts of milk proteins (Fig. 4B). Blotting with a specific anti-β-casein antibody confirmed the reduced amount of this milk protein in the RAB6A-depleted tissue samples analyzed (Fig. 4C). Immunolocalization studies showed that alveolar luminal cells from the control tissue homogeneously displayed β-casein at their apical pole. By contrast, mutant alveolar luminal cells expressed β-casein at low level and/or exhibited a poorly polarized distribution pattern (Fig. 4D).

In addition to proteins and lipids, lactose is an essential milk component. Its massive synthesis from glucose during lactation is accompanied by the upregulated expression of the glucose transporter GLUT1 at the baso-lateral membrane of alveolar luminal cells (Boxer et al., 2006; Anderson et al., 2007). WB analysis at L1 revealed a reduced amount of GLUT1 in the RAB6A-depleted mammary samples compared with the controls (Fig. 4E). As expected, GLUT1 was homogeneously expressed in the control tissue and sharply localized at the baso-lateral membrane of the alveolar luminal cells (Fig. 4F). By contrast, in mutant alveolar luminal cells, GLUT1 displayed a disorganized expression pattern; it was absent from the lateral cell membranes and irregularly expressed basally (Fig. 4F). Other polarity markers, including baso-lateral (E-cadherin, β1 integrin) and apical (ZO-1, MUC1) markers, appeared to be normally distributed in RAB6A-depleted luminal cells (Fig. S4A-C).

Collectively, our data revealed a postpartum failure in secretory activation of alveolar luminal cells. The reduced milk protein production, CLD retention and decreased GLUT1 expression suggest that milk amount and composition might be affected in the absence of RAB6A, compromising pup growth and viability.

### *Rab6a* loss in alveolar luminal cells affects RAB18 expression

**Fig. 5.**
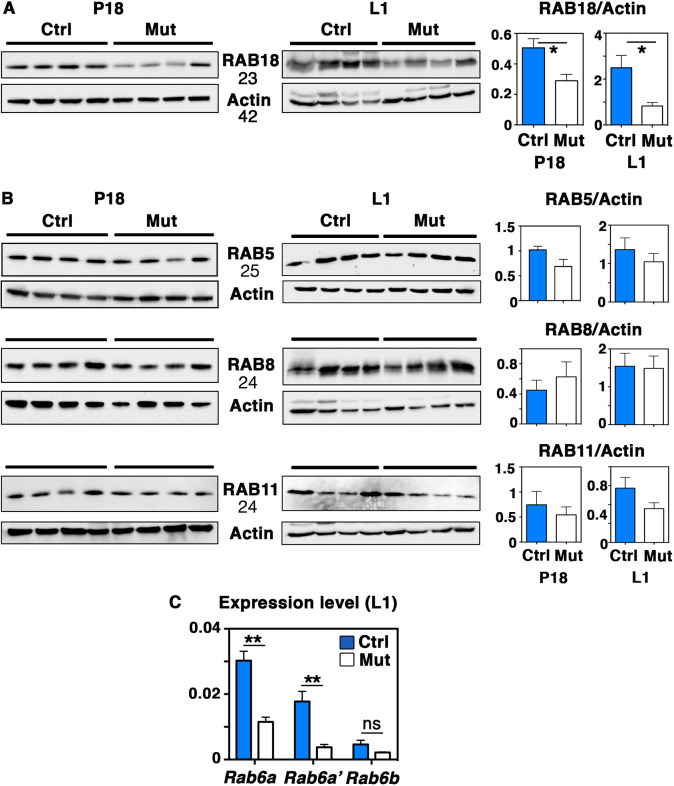
**RAB expression in *Rab6a*-deficient mammary glands.** (A) Western blots for RAB18 and actin at P18 and L1 (left panels) and quantifications (mean±s.e.m.) normalized on actin amounts (right panels). Data are from four distinct control and mutant mammary gland protein extracts at P18 and L1. *P=0.029 (P18), *P=0.048 (L1). (B) Western blots for RAB5, RAB8, RAB11 at P18 and L1 and their actin loading controls (left panels). The samples are the same as those shown in A. The related quantifications shown on the right panels did not reveal any significant differences between control and mutant samples. (C) qPCR analysis of *Rab6a*, *Rab6a*′ and *Rab6b* mRNA expression in mammary cells derived from control and mutant glands at L1. qPCR values were normalized on *Gapdh*. Data are mean±s.e.m. of four separate cell preparations. **P=0.001 (*Rab6a*), **P=0.005 (*Rab6a′*). ns, not significant. MW are indicated in kD.

As described above, alveolar luminal cells from BlgCre; *Rab6a*^F/F^ females display deficient CLD maturation and apical targeting (Figs 2E and 4A). RAB18 has been reported to control CLD growth and secretion in various lipogenic cell types (Xu et al., 2018; Dejgaard and Presley, 2019). Consistently, RAB18 is one of the RAB GTPases that is highly expressed in mammary luminal cells (Fig. 1B). We thus examined whether *Rab6a* loss affected RAB18 expression. Using WB, we found that RAB18 amounts were significantly decreased in the protein extracts from P18 and L1 mutant glands, compared with controls (Fig. 5A). Noticeably, the amount of other abundantly expressed RAB GTPases (RAB5, RAB8 and RAB11) was not significantly altered in the mutant samples (Fig. 5B), indicating that *Rab6a* loss had a particular impact on RAB18.

In addition, qPCR data showed that *Rab6a*-deleted and control mammary glands displayed similar low levels of *Rab6b* at L1 (Fig. 5C). Thus, *Rab6b* remained poorly expressed at the onset of lactation and was not significantly affected upon *Rab6a* deletion, making compensatory mechanisms by RAB6B unlikely.

### *Rab6a* loss in alveolar luminal cells leads to diminished STAT5 activation

**Fig. 6.**
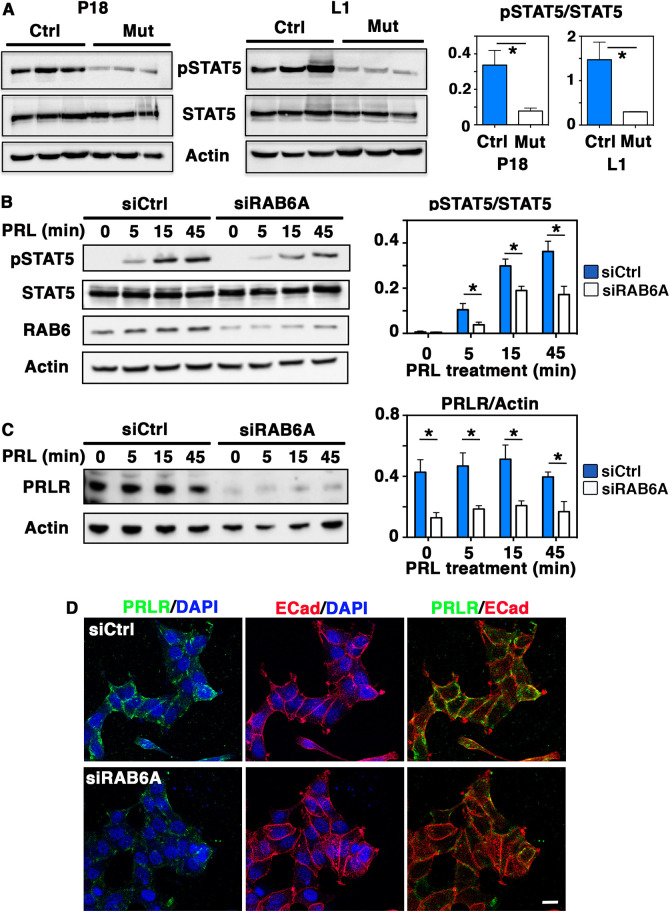
**Decreased STAT5 activation in *Rab6a*-deficient mammary glands and RAB6A-depleted T47-D cells.** (A) Western blots for pSTAT5, total STAT5 and actin performed on three distinct control and mutant mammary gland protein extracts at P18 and L1 (left). Quantification of pSTAT5/STAT5 amounts (mean±s.e.m.) (right). *P=0.038 (P18), *P=0.041 (L1). (B) Western blots for pSTAT5, total STAT5, RAB6 and actin performed with T47-D cell lysates (left). SiCtrl and SiRAB6A cells were stimulated with PRL for 5, 15 and 45 min. Quantification of pSTAT5/STAT5 amount at each time point (right). Data are mean±s.e.m. of four separate siRNA assays. *0.01<*P*<0.05. (C) Western blots for PRLR and actin performed with the same T47-D cell lysates as in B (left). Quantifications of PRLR amount normalized on actin at each time point (right). Data are mean±s.e.m. of three separate siRNA assays. *0.01<*P*<0.05. MW are indicated in kD. (D) Immunolocalization of PRLR and E-cadherin (ECad) in unstimulated SiCtrl and SiRAB6A T47-D cells. Nuclei are stained with DAPI. Images are a *z*-projection of 15 confocal planes across the cells. Scale bar: 20 µm.

The transcription factor STAT5 plays a key role in secretory differentiation and activation of the alveolar epithelium, in particular downstream of PRLR binding to PRL (Hennighausen and Robinson, 2008). To gain mechanistic insight into the defects observed in BlgCre; *Rab6a*^F/F^ mammary glands, we analyzed levels of the activated phosphorylated form of STAT5 (pSTAT5) in the protein extracts obtained at P18 and L1. WB data showed that, compared with controls, mutant samples displayed a marked decrease in the ratios between pSTAT5 and total STAT5 amounts, indicating a reduced STAT5 activation upon *Rab6a* deletion (Fig. 6A). Expression of ELF5, a transcription factor controlling alveolar luminal cell maturation downstream of PRL signaling together with STAT5 (Lee and Ormandy, 2012), was also diminished in mutant samples at L1 (Fig. S4D). On the other hand, similar levels of phosphorylated focal adhesion kinase (pFAK; PTK2) were observed in mutant and control samples at L1 (Fig. S4D). This indicated that integrin signaling, known to cooperate with PRL signaling for optimal STAT5 activation (Akhtar et al., 2009), was not critically affected upon *Rab6a* deletion.

To investigate the possible molecular mechanisms underlying the reduced STAT5 activation observed upon *Rab6a* deletion, we took advantage of T47-D, a human breast cancer-derived cell line commonly used for studying PRL-induced signaling events (Johnson et al., 2010; Baker et al., 2016; Oakes et al., 2017). Double staining for RAB6 and GM130 confirmed the expected localization of RAB6 in the Golgi of T47-D cells (Fig. S5A). RAB6A depletion was achieved using siRNAs targeting *Rab6a/a*′ variants, as previously reported (Patwardhan et al., 2017). WB analysis of protein extracts showed that siRAB6A T47-D cells contained half of the RAB6 amount found in siCtrl T47-D cells (Fig. 6B and Fig. S5B). Immunofluorescence labeling confirmed the depletion of RAB6 in siRAB6A T47-D cells (Fig. S5C).

STAT5 being rapidly activated upon PRL binding, we analyzed T47-D cell response after 5, 15 and 45 min of PRL stimulation. Notably, at each time point, we observed a marked decrease in the pSTAT5/STAT5 ratios in RAB6A-depleted T47-D cells compared with non-depleted cells (Fig. 6B). To examine at which level PRL signaling was affected in this model, we analyzed PRLR expression using an antibody recognizing the PRLR extracellular domain. Data from WB showed that RAB6A-depleted T47-D cells contained about half the level of PRLR than non-depleted cells and that the decreased PRLR amount persisted throughout PRL stimulation (Fig. 6C). Immunofluorescence studies indicated that most control T47-D cells displayed PRLR at their cell-cell contacts and borders, revealed by E-cadherin labeling (Fig. 6D and Fig. S5D). This expression pattern largely disappeared in RAB6A-depleted cells that overall exhibited a weak PRLR expression (Fig. 6D and Fig. S5C,D), as expected from the WB data. Unlike PRLR, E-cadherin expression was not affected by RAB6A depletion (Fig. 6D and Fig. S5D).

Collectively, the results obtained with the mammary tissue and the T47-D cell line revealed that RAB6A depletion led to diminished STAT5 activation. In addition, they suggested that this defect probably occurred through an altered expression of PRLR at the cell membrane, resulting in decreased PRL-induced signaling events.

## DISCUSSION

Our *in vivo* study uncovers a role for RAB6A in the differentiation and maturation of the luminal secretory lineage. Loss of *Rab6a* severely compromised the lactational function of the gland and the maintenance of the secretory tissue. It led to a decreased activation of STAT5, a key downstream effector of PRL-induced lactational processes. Data obtained with the PRL-responding human mammary cell line T47-D showed that loss of *Rab6a* led to decreased expression of PRLR, suggesting a perturbed transport of PRLR altering its surface expression and consequently its signaling cascade. These defects might largely account for the mammary phenotype observed in BlgCre; *Rab6a*^F/F^ females. Noticeably, despite the major functional deficiency of the RAB6A-depleted alveolar luminal cells, the polarized organization of the mammary bilayer was preserved *in vivo*.

### Loss of *Rab6a* in the secretory lineage has no major impact on mammary morphogenesis during gestation

To delete *Rab6a* in the secretory lineage we used the *Blg* promoter, known to be specifically active in the HR^−^ luminal progenitors and their secretory progeny from mid-pregnancy. This promoter is inactive in the basal and HR^+^ luminal cell lineages, throughout mammary development (Selbert et al., 1998; Chapman et al., 1999; Naylor et al., 2005; Molyneux et al., 2010; Di-Cicco et al., 2015; Romagnoli et al., 2020). The mammary epithelium expands massively until late pregnancy. During this morphogenetic period, the HR^−^ luminal and basal progenitors are amplified, primarily through progesterone and local paracrine mediators secreted by the hormone-responsive HR^+^ cells (Fata et al., 2000; Asselin-Labat et al., 2010; Brisken and Ataca, 2015; Yu et al., 2016). Whole-mount and flow cytometry analyses did not reveal any severe underdevelopment of BlgCre; *Rab6a*^F/F^ mammary glands or perturbation of the luminal-to-basal cell balance until late gestation. This indicates that basal and luminal progenitors have proliferated actively upon *Rab6a* deletion. Interestingly, unlike the control, the mutant epithelium was still actively proliferating at late gestation (P18), suggesting that loss of *Rab6a* may lead to delayed mammary development, possibly associated with an accumulation of HR^−^ luminal progenitors and perturbed differentiation events.

### RAB6A plays a crucial role in the differentiation, activation and maintenance of the luminal secretory cells

The major defects induced by loss of *Rab6a* clearly appeared at late gestation during the prelactogenic differentiation period and postpartum with the secretory activation. This led to a lactation deficiency in primiparous females, revealing a crucial role for RAB6A in mammary gland function.

Mammary glands from numerous transgenic models showing deficiency in milk protein and lipid production display poor alveolar distension associated with defective alveolar luminal cell maturation (Naylor et al., 2005; Li et al., 2005; Russell et al., 2011; Morales et al., 2012; Akhtar et al., 2016). This phenotype was observed in BlgCre; *Rab6a*^F/F^ at P18 and L1. Several aspects of the luminal secretory cell maturation were perturbed, in particular the global production of milk proteins, the expansion of CLDs and their apical targeting, and the baso-lateral expression of the glucose transporter, GLUT1. Conceivably, these perturbations altered both milk quantity and composition, compromising pup survival.

Loss of *Rab6a* led to a precocious luminal cell death as early as postpartum, showing that RAB6A is essential for the maintenance of the secretory tissue. The precise mechanisms remain to be determined; however, it is known that the secretory differentiation and activation steps are essential to trigger and maintain the lactation process (Fata et al., 2000; Vorbach et al., 2002; Cui et al., 2004; Anderson et al., 2007; Russell et al., 2011). When these steps are compromised, luminal cells die as observed during mammary gland involution, a physiological process that normally occurs after weaning (Watson and Kreuzaler, 2011).

### Loss of *Rab6a* does not critically affect the polarized organization of the mammary bilayer

Loss of RAB6A during mouse embryogenesis and in migratory cell models has been reported to severely alter laminin and/or β1 integrin expression, perturbing cell adhesion and migration (Shafaq-Zadah et al., 2016). In MDCK cells, which present an apico-basal polarity when grown in 3D cultures, RAB6A/B knock-out (RAB6-KO) strongly inhibited the secretion of soluble cargos including laminin, whereas it only mildly impaired that of transmembrane proteins (Homma et al., 2019). Of note, despite their lack of laminin secretion, RAB6-KO MDCK cells were able to form polarized cysts in 3D cultures.

We did not detect any obvious perturbation of laminin deposition in the mammary epithelium from BlgCre; *Rab6a*^F/F^ females at both late gestation and during lactation. In the mammary bilayer, basal myoepithelial cells rather than luminal cells express a wide range of genes coding for extracellular matrix proteins (Kendrick et al., 2008; Lim et al., 2010). It is therefore considered that basal cells, together with the surrounding stromal cells, produce most of the basement membrane components of the mammary epithelium (Moumen et al., 2011; Glukhova and Streuli, 2013).

Although less enriched in integrins than basal cells, luminal cells display several integrin chains, including α6 and β1 chains (Glukhova and Streuli, 2013; Romagnoli et al., 2019, 2020). Our data did not reveal notable perturbations in α6 and β1 chain expression or in FAK activation in RAB6A-deficient luminal cells. Consistently, the mammary phenotype of BlgCre; *Rab6a*^F/F^ females did not phenocopy that observed in BlgCre; *Itgb1*^F/F^, BlgCre; *Itga3;Itga6*^F/F^ and BlgCre; *Ilk*^F/F^ mice (Naylor et al., 2005; Akhtar and Streuli, 2013; Romagnoli et al., 2020). In these models, during lactation, alveoli displayed luminal cell clusters protruding into the lumen, a phenotype absent upon *Rab6a* deletion.

Several essential markers of the apico-basal polarity, such as E-cadherin, MUC1 and ZO-1, appeared properly located to the cell surface of RAB6A-deficient alveolar luminal cells. Moreover, GM130 immunolabeling did not reveal mis-localization of the Golgi, a characteristic observed in BlgCre; *Itgb1*^F/F^, BlgCre; *Itga3;Itga6*^F/F^ and BlgCre; *Ilk*^F/F^ alveolar luminal cells that displayed perturbed apico-basal polarity (Akhtar and Streuli, 2013; Romagnoli et al., 2020).

Potentially, other RAB proteins, highly expressed in the luminal secretory cells, could participate in maintaining apico-basal polarity features, in particular RAB8, a RAB GTPase implicated in the same post-Golgi transport pathways as RAB6A (Goud and Akhmanova, 2012). In addition, although expressed at very low level in the mammary tissue, RAB6B could have some compensatory function in the absence of RAB6A, as reported for RAB6-KO MDCK cells (Homma et al., 2019).

### Loss of *Rab6a* led to a decreased activation of STAT5 in the secretory tissue

STAT5 is a key transcription factor controlling the mammary lobulo-alveolar development and the lactogenic function of the secretory lineage. Its activation level is strictly regulated in luminal cells: it gradually increases from mid- to late gestation and remains high throughout lactation (Hennighausen and Robinson, 2008; Hughes and Watson, 2012). Noticeably, we observed a decreased STAT5 activation in BlgCre; *Rab6a*^F/F^ mammary glands at P18 and L1.

Hennighausen and colleagues have shown that STAT5 controls, directly or indirectly, the expression of multiple milk protein genes and regulatory molecules involved in the lactation process (Yamaji et al., 2013). These include, in addition to the β-casein and Wap genes, *Adph* required for CLD maturation in the mammary tissue and *Rab18* implicated in CLD maturation and transport in lipogenic cells (Russell et al., 2011; Dejgaard and Presley, 2019). Interestingly, we found that *Rab6a* loss affected RAB18 expression but not other highly expressed RAB proteins, suggesting a functional molecular link between RAB6 and RAB18. Such a link could involve the ZW10 and RINT1 proteins, which are part of the NRZ (NAG-RINT1-ZW10) complex, a RAB18 effector that tethers CLDs to membranes of the endoplasmic reticulum (Xu et al., 2018). On the other hand, RAB6 regulates a Golgi-to-endoplasmic retrograde transport pathway through a ZW10/RINT1 complex (Sun et al., 2007).

STAT5 also controls lactose production by inducing the expression of α-lactalbumin and numerous members of the solute carrier family, such as *Slc2a1* encoding GLUT1 (Yamaji et al., 2013). In addition, perturbation of STAT5 activation negatively impacts the expression of ELF5, a transcription factor cooperating with STAT5 in the specification of the secretory lineage (Lee and Ormandy, 2012; Yamaji et al., 2013). Milk proteins, ELF5, ADPH, GLUT1 and RAB18 were found to display reduced or altered expression in BlgCre; *Rab6a*^F/F^ mammary glands at P18 and/or L1, indicating that deregulated STAT5 activation might largely account for the perturbed function of the RAB6A-deficient secretory tissue.

### Loss of *Rab6a* affects PRLR expression and its downstream STAT5 signaling

STAT5 is predominantly activated by PRL, a pituitary hormone that plays a major role during the pre-lactogenic and lactation periods (Hennighausen and Robinson, 2008; Hughes and Watson, 2012). Therefore, the decreased STAT5 activation observed at these stages in the mammary tissue from BlgCre; *Rab6a*^F/F^ females strongly evokes perturbation in the PRL/PRLR signaling events.

Studies on PRLR signaling in the mouse mammary gland have been hampered by the lack of reliable antibodies working in immunohistochemistry, immunocytochemistry or immunoblotting (Brisken and Ataca, 2015). To circumvent this difficulty, we used the well-characterized PRL-responding human T47-D cells (Johnson et al., 2010; Baker et al., 2016; Oakes et al., 2017). Using siRNAs, we found that RAB6A depletion in this model resulted in decreased PRLR levels, altered membrane expression and reduced STAT5 activation downstream of PRL action. RAB6A plays a role in all steps of post-Golgi secretion (Goud and Akhmanova, 2012; Fourriere et al., 2019). Loss of RAB6A might thus perturb PRLR transport at different levels from Golgi to the plasma membrane, affecting its surface expression and thereby PRL-induced signaling events. Whether perturbation of intracellular PRLR transport leads to PRLR delivery to lysosomes for degradation, as reported for unsecreted cargos in RAB6-KO MDCK cells (Homma et al., 2019), remains to be determined.

Our study reveals for the first time a role for RAB6A in the lactogenic function of the mammary luminal secretory lineage and suggests that RAB6A controls STAT5 activation by regulating PRLR membrane expression. The level of PRLR expressed by mammary luminal cells is known to be crucial for proper alveolar development and lactation, as observed in a mouse model containing a single *Prlr* functional allele and displaying haploinsufficiency (Ormandy et al., 1997; Hennighausen and Robinson, 2008). In addition, high levels of STAT5 activation are required for luminal secretory cells to proceed through a full differentiation program (Yamaji et al., 2013).

These conclusions do not exclude the possibility that *Rab6a* loss induced various trafficking defects for proteins other than PRLR. In particular, this might be the case for milk proteins, CLD-associated proteins and GLUT1 that all displayed a perturbed localization in the RAB6A-deficient luminal secretory cells. Further investigations are needed to decipher these dynamic intracellular aspects, using relevant *in vitro* models such as mammary organoids and milk-producing cell lines.

Finally, it is worth adding that RAB6A has been implicated in matrix protein secretion, cell-matrix interactions and cell polarity in different models (Shafaq-Zadah et al., 2016; Fourriere et al., 2019; Homma et al., 2019). By contrast, our data indicate that loss of *Rab6a* in the luminal secretory lineage had no major impact on the structure of the mammary tissue. RAB6A function therefore appears to depend on cell type specialization and tissue context. Conceivably, in polarized tissues, distinct polarity features – apico-basal, front-rear and dual – might influence RAB6A-dependent intracellular trafficking pathways.

## MATERIALS AND METHODS

### Mouse strains and transgenic mice

BlgCre transgenic mice, expressing the Cre recombinase under the control of the *Blg* promoter, were purchased from The Jackson Laboratory. They were bred in a mixed CBA/C57Bl6 background. *Rab6a*^F/F^ mice (99% C57Bl6 background) have been previously characterized (Bardin et al., 2015). Animal care and use for this study were performed in accordance with the recommendations of the European Community (2010/63/UE) for the care and use of laboratory animals. Experimental procedures were specifically approved by the ethics committee of the Institut Curie CEEA-IC #118 (Authorization APAFiS# 26880-20200813165686-v1 given by National Authority) in compliance with the international guidelines.

### Dissociation of mouse mammary glands and flow cytometry analysis

Single cells were prepared from a pool of thoracic and inguinal mammary glands harvested from adult females (12-week-old virgin or 15- and 18-day-pregnant), as described in detail elsewhere (Di-Cicco et al., 2015). Briefly, minced tissues were transferred to a digestion solution containing 3 mg/ml collagenase (Roche), 100 units/ml hyaluronidase (Sigma-Aldrich) in CO_2_-independent medium (Gibco Life Technologies) completed with 5% fetal bovine serum (FBS; Lonza) and 2 mM L-glutamine (Sigma-Aldrich), and incubated for 90 min at 37°C with shaking. Pellets of digested samples were centrifuged (450 ***g***) and successively treated at 37°C with solutions of 0.25% trypsin (Life Technologies)/0.1% versen (Biochrom) for 1 min and 5 mg/ml dispase II (Roche)/0.1 mg/ml DNAseI (Sigma-Aldrich) for 5 min. Pellets were treated with a cold ammonium chloride solution (Stem Cell Technologies) and filtered through a nylon mesh cell strainer with 40 µm pores (Fisher Scientific) before immunolabeling.

Freshly isolated mammary cells were incubated at 4°C for 20 min with the following antibodies from Biolegend: anti-CD24-BViolet421 (clone M1/69; #101826; 1/50), anti-CD49f-PeCy7 (clone GoH3; #313622; 1/50), anti-CD29-PeCy7 (clone HMβ1-1; #102222; 1/100), anti-CD45-APC (clone 30-F11; #103112; 1/100), anti-CD31-APC (clone MEC13.3; #102510; 1/100), anti-CD54-PE (clone YN1/1.7.4; #116107; 1/50). Labeled cells were analyzed and sorted using a MoFlo Astrios cell sorter (Beckman Coulter). Data were analyzed using FlowJo software. Sorted cell population purity was at least 95%.

### Microarray data analysis of *Rab* expression

Global gene expression analysis was performed with total RNA extracted from luminal cell populations isolated by flow cytometry as previously reported (Chiche et al., 2019; https://www.ncbi.nlm.nih.gov/geo/query/acc.cgi?acc=GSE122928). Samples were hybridized on Affymetrix GeneChip Mouse Genome 2.1ST arrays. Hierarchical clustering was performed using hclust (R) with Euclidean distance and Ward agglomeration method. GTPase genes were classified according to their expression level that was considered significant if >7.

### Whole-mount analyses and histology

Dissected mammary fat pads were spread onto glass slides, fixed in Methacarn (1/3/6 mixture of acetic acid/chloroform/methanol) overnight at room temperature and stained with carmine alum (Stem Cell Technologies) or fixed in 4% paraformaldehyde overnight at 4°C, as previously described (Bresson et al., 2018). For histological analyses, fixed glands were embedded in paraffin, and 7-μm-thick sections were cut, dewaxed and stained with H&E. Image acquisition was performed using Nikon Eclipse 90i Upright microscope. ImageJ software (National Institutes of Health) was used to quantify fat pad occupancy and alveolar size. Frozen sections (5 or 8 μm thick) were obtained after embedding mammary glands into Tissue-Tek (Sakura), using a Leica Cryostat. For RAB6 immunodetection, mammary glands were fixed in 4% paraformaldehyde overnight at 4°C, and after incubation time in sucrose solutions were embedded into Tissue-Tek before freezing.

### Immunohistofluorescence labeling

Paraffin sections were dewaxed, processed for acidic antigen retrieval, incubated overnight at 4°C with primary antibodies, and then at room temperature with secondary antibodies for 2 h. Frozen sections fixed with 4% paraformaldehyde or acetone were incubated with antibodies as paraffin sections.

The following primary antibodies were used: anti-K5 and anti-K8 (Biolegend; #905501; RRID: AB_2565050; 1/1000 and #904801; RRID: AB_2565043; 1/100, respectively), anti-αSMA (Sigma Aldrich; #A2547; RRID: AB_476701; 1/200), anti-PR (Santa Cruz; #sc-7208; RRID: AB_2164331; 1/200), anti-Ki67 (Thermo Fisher Scientific; #MA5-14520; RRID: AB_10979488; 1/100), anti-pan-laminin (Abcam; #ab11575; RRID: AB_298179; 1/100), anti-ZO-1 (Thermo Fisher Scientific; #61-7300; RRID: AB_2533938; 1/200), anti-MUC1 (Abcam; #ab37435; RRID: AB_776551; 1/200), anti-E-cadherin ECCD-2 (Life Technology; #13-1900; RRID: AB_86571; 1/200), anti-adipophilin (Progen; #GP40; 1/100), anti-GLUT1 (Abcam; #40084; RRID: AB_2190927; 1/50), anti-β1 integrin (Millipore; MAB1997; RRID: AB_2128202; 1/100), anti-GM130 (BD Biosciences; #610823; RRID: AB_398142; 1/100), anti-RAB6 (Santa Cruz Biotechnology; #sc-310; RRID: AB_2175466 1/100). The anti-mouse β-casein was designed by Covalab.

Alexafluor-488 or Cy3 conjugated secondary antibodies were from Jackson ImmunoResearch Laboratories (709-165-149, 715-165-150, 711-465-152, 712-165-153, 709-545-149, 715-545-151, 711-545-152, 712-545-153, 706-165-148; 1/200). Immunostained tissue sections were mounted in Prolong Gold antifade reagent with DAPI (Invitrogen, Life Technologies).

Images from Figs 1D and 3B were taken using a Nikon confocal A1R microscope (60× oil objective). Images from Figs 2G, 4D,F were acquired using a Zeiss LSM880 NLO inverted laser scanning confocal with an Airyscan module (63× oil objective). Images from Fig. 6D and Fig. S5C,D were taken using a Leica SP8 NLO inverted laser scanning confocal microscope (40× oil objective). Other images were obtained using a conventional epifluorescence microscope (Leica DM 6000B; 20× dry and 40× oil objectives) equipped with MetaMorph software.

### TUNEL assay

For cell death analysis, glands were fixed in 4% paraformaldehyde in PBS (pH 7.5) overnight at 4°C. Dewaxed paraffin sections were analyzed for TdT digoxygenin nick-end labeling with Apoptag Plus (Sigma Aldrich) following the manufacturer's instructions. Methyl green was used as counterstaining. Image acquisition was performed using a Leica DM RBE optic microscope and the number of TUNEL^+^ cells per field was counted using ImageJ software.

### RNA extraction and qPCR

RNA was isolated from whole mammary glands using Trizol reagent (Life Technologies) and further purified on a cleanup column (Qiagen). RNeasy Microkit was used for RNA extraction from isolated mammary cells, as previously described (Di-Cicco et al., 2015; Chiche et al., 2019). To avoid eventual DNA contamination, purified RNA was treated with DNAse (Qiagen). RNAs were reverse-transcribed using MMLV H(-) Point reverse transcriptase (Promega). Quantitative PCR was performed using the QuantiNova SYBR Green PCR Kit (Qiagen) on a LightCycler 480 real-time PCR system (Roche). The values obtained were normalized to *Gapdh* levels. The primers used for qPCR analysis were purchased from SABiosciences/Qiagen or designed using Oligo 6.8 software (Molecular biology Insights) and synthesized by Sigma Aldrich. Primers for *Rab6a*, *Rab6a*′ and *Rab6b* used in this study are listed in Table S2.

### T47-D cell culture and RAB6A knockdown by siRNA

The human mammary epithelial cell line T47-D was kindly provided by Dr V. Goffin (INSERM UMR_S1151-CNRS UMR8253, Institut Necker-Enfants Malades, Paris, France) and tested for contamination. Cells were maintained at 5% CO_2_ in a 37°C incubator and grown in a phenol red-free RPMI 1640 medium containing 10% FBS and 5 µg/ml insulin, as previously described (Baker et al., 2016).

For siRNAs knockdown experiments, cells were transfected the day after their seeding with siRNA controls (siLuciferase) or specific siRAB6A/A′ (50 nM final concentration) in Lullaby reagent (OZ Biosciences). A second transfection was performed 24 h after the first one. Experiments were conducted 72 h after the last transfection.

Cells were washed in PBS 24 h before PRL treatment and medium was replaced by a serum-free medium. Cells were treated with human PRL (Sigma Aldrich, #L4021) at 250 ng/ml for 5, 15 and 45 min at 37°C in a medium supplemented with 5 µg/ml insulin and 1 µM dexamethasone, as previously described (Baker et al., 2016).

Immunofluorescence staining of PRLR (anti-PRLR, Invitrogen; #35-9200; 1/200) and RAB6 (AC306, produced and purified in B.G.’s lab; 1/1000; Goud et al., 1990) were performed after fixing the cells with 4% paraformaldehyde and mild permeabilization with 0.05% saponin.

### Western blot analysis

Mammary tissue samples were homogenized in RIPA extraction buffer [0.1% SDS, 276 mM NaCl, 40 mM Tris (pH 7.5), 2% NP-40, 4 mM EDTA, 20% glycerol, 1× protease inhibitors] following further incubation for 20 min at 4°C on a rotation wheel. After centrifugation at 12,000 rpm (12,800 ***g***) for 15 min at 4°C, supernatants containing extracted proteins were recovered and the BCA Protein assay kit from Pierce (#23225) was used to estimate protein concentration. Before migration, 40 μg of protein extracts were boiled for 5 min in Laemmli buffer. Cell pellets were resuspended in 1× Laemmli buffer, vortexed and boiled for 5 min. Samples were run on NuPAGE Novex 4-12% Bis Tris gels (Life Technologies/Invitrogen) and transferred onto nitrocellulose. Membranes were incubated with 5% bovine serum albumin in TBS containing 0.1% Tween 20 (TBST) for 1 h at room temperature and with primary antibodies overnight at 4°C.

The following primary antibodies were used on mouse cell-derived lysates: anti-mouse milk proteins (Accurate Chemical; YNRMMSP; 1/2000), anti-mouse β-casein (a kind gift from C. Streuli, Manchester University; 1/1000), anti-RAB6 (AC306, produced and purified in B.G.'s lab; 1/2000), anti-RAB5 [Cell Signaling Technology (CST); #3547S; RRID: AB_2300649; 1/1000], anti-RAB8 (BD Biosciences; #610844; RRID: AB_398163; 1/1000), anti-RAB11 (BD Biosciences; #610656; RRID: AB_397983; 1/1000), anti RAB18 (Sigma Aldrich; #SAB4200173; RRID: AB_10638775; 1/1000), anti-GLUT1 (Abcam; #40084; RRID: AB_2190927; 1/500), anti-ELF5 (Santa Cruz Biotechnology; sc-9645; RRID: AB_640106; 1/500), anti-STAT5 (Santa Cruz Biotechnology; #sc-1081; RRID: AB_632448; 1/10,000), anti-pSTAT5-Tyr694 (CST; #9359; RRID: AB_823649; 1/1000), anti-pFAK-Tyr397 (CST; #3283S; RRID: AB_2173659; 1/750), anti-FAK (CST; #3285; RRID: AB_2269034; 1/1000). T47-D cell lysates were probed using anti-RAB6 (AC306, produced and purified in B.G.'s lab; 1/2000), anti-STAT5 (BD Biosciences; #610191; RRID: AB_397590; 1/1000), anti-pSTAT5-Tyr694 (CST; #9359; RRID: AB_823649; 1/1000) and anti-PRLR (Invitrogen; #35-9200; RRID: AB_2533231; 1/500).

Secondary antibodies coupled to horseradish peroxidase were from Jackson ImmunoResearch Laboratories (711-035-152, 715-035-150, 705-035-147; 1/10,000). Detection was performed by chemiluminescence (Super signal West Pico+ or Femto, Thermo Fisher Scientific). Quantitative analysis was performed using ImageLab*.*

### Statistical analysis

*P*-values were determined using unpaired Student's *t-*test with two-tailed distribution and Welch's correction, using the GraphPad prismv6 software. Different numbers of asterisks indicate differing levels of significance: *P<0.05, **P<0.01, ***P<0.0001. When specified, a Pearson's Chi-square test was applied. Data are mean±s.e.m.

## Supplementary Material

Supplementary information
