# Supplementary material for: RAB6 GTPase regulates mammary secretory function by controlling the activation of STAT5
Source: Development. 2020 Oct 8;147(19):dev190744. doi: 10.1242/dev.190744 (PMC7561474; doi:10.1242/dev.190744)
Supplement: Supplementary information [file develop-147-190744-s1.pdf]

SUPPLEMENTARY MATERIALS

TABLES

Table S1. ***Rab* gene expression in the ER$^+$/PR$^+$ and ER$^-$/PR$^-$ mammary luminal cell populations isolated from adult virgin females using ICAM-1 §**

	**HR$^+$ cells (CD24^{high} ICAM$^-$)**		**HR$^-$ cells (CD24^{high} ICAM$^+$)**	
Gene symbol	**Mean**	SEM	**Mean**	SEM
Rab1a	**10.248**	0.061	**10.398**	0.046
Rab1b	**7.839**	0.108	**7.742**	0.232
Rab2a	**9.160**	0.062	**9.123**	0.114
Rab2b	**5.803**	0.099	**5.906**	0.193
Rab3a	**5.789**	0.105	**5.613**	0.179
Rab3b	**4.556**	0.135	**4.772**	0.078
Rab3c	**4.281**	0.038	**4.263**	0.083
Rab3d	**6.267**	0.124	**5.955**	0.118
Rab4a	**8.343**	0.118	**8.535**	0.146
Rab4b	**7.448**	0.162	**6.644**	0.120
Rab5a	**7.406**	0.148	**7.299**	0.151
Rab5b	**6.405**	0.108	**6.067**	0.098
Rab5c	**8.299**	0.069	**8.174**	0.110
Rab6a	**7.534**	0.180	**7.582**	0.183
Rab6b	**4.987**	0.326	**5.155**	0.329
Rab7a	**9.586**	0.055	**9.680**	0.067
Rab7b	**6.163**	0.091	**7.093**	0.097
Rab8a	**7.520**	0.137	**7.321**	0.137
Rab8b	**7.322**	0.125	**7.694**	0.162
Rab9a	**7.695**	0.080	**7.982**	0.173
Rab9b	**5.204**	0.084	**5.417**	0.161
Rab10	**9.152**	0.103	**9.270**	0.071
Rab11a	**8.672**	0.117	**8.317**	0.072
Rab11b	**8.338**	0.147	**8.142**	0.139
Rab12	**6.399**	0.131	**6.600**	0.205
Rab13	**6.716**	0.172	**6.185**	0.248
Rab14	**8.811**	0.061	**8.857**	0.073
Rab15	**6.444**	0.105	**6.273**	0.244
Rab17	**5.407**	0.264	**5.817**	0.255
Rab18	**7.922**	0.204	**8.095**	0.117
Rab19	**4.967**	0.211	**4.969**	0.147
Rab20	**6.458**	0.202	**6.388**	0.252
Rab21	**8.098**	0.101	**8.363**	0.125
Rab22a	**6.303**	0.081	**6.219**	0.116
Rab23	**5.987**	0.128	**5.867**	0.189

Development • Supplementary information

Rab24	**7.975**	0.092	**7.805**	0.175
Rab25	**8.583**	0.114	**8.299**	0.152
Rab26	**4.990**	0.103	**4.818**	0.075
Rab27a	**5.384**	0.108	**5.036**	0.135
Rab28	**6.149**	0.159	**5.871**	0.224
Rab29	**6.039**	0.210	**6.217**	0.191
Rab30	**4.830**	0.097	**5.752**	0.176
Rab31	**5.963**	0.157	**4.983**	0.210
Rab32	**4.877**	0.178	**6.077**	0.225
Rab33a	**4.626**	0.094	**4.819**	0.108
Rab33b	**7.079**	0.128	**6.641**	0.183
Rab34	**5.777**	0.179	**6.100**	0.243
Rab35	**5.959**	0.073	**5.978**	0.130
Rab36	**5.696**	0.138	**5.915**	0.165
Rab37	**5.208**	0.142	**4.426**	0.104
Rab38	**5.963**	0.274	**5.326**	0.106
Rab39	**4.126**	0.065	**4.279**	0.074
Rab39b	**4.694**	0.051	**4.645**	0.067
Rab40b	**4.444**	0.085	**4.630**	0.209
Rab40c	**5.541**	0.094	**5.999**	0.125
Rab42	**4.772**	0.097	**4.690**	0.128
Rab43	**4.704**	0.077	**4.818**	0.173
Elf5	**6.751**	0.235	**9.345**	0.089
Icam1	**6.670**	0.250	**7.832**	0.201
Esr1	**8.218**	0.142	**7.177**	0.240
Pgr	**7.112**	0.140	**5.711**	0.180

§ Data are from a previously published microarray analysis, available on https://www.ncbi.nlm.nih.gov/geo/ query/acc.cgi?acc=GSE122928 (Chiche et al., 2019). The expression values (normalized Log_2 gene expression level) are shown as mean ± SEM. Seven distinct mammary cell preparations and cell sorting experiments were analyzed. Each distinct cell preparation was obtained from a pool of mammary glands taken from 3-4 females. As in the heat map shown in Fig. 1B, robust expression levels > 7 appear in red whereas levels ≤7 appear in blue. Reference genes (*Icam1*, *Elf5*, *Esr1*, *Pgr*) used to discriminate the luminal cell populations are listed at the end of the table, with highest expression levels in red.

Development • Supplementary information

Table S2: Primers for qPCR

qPCR primers	Sequence 5'-3'
Rab6a-F<br>Rab6a-R	GCC TCA TTC CCA GTT ACA TCC<br>TCC ATT TTG TAG TTT GCT GGA A
Rab6a'-F<br>Rab6a'-R	AAA CAA TGT ACT TGG AGG ATA GAA CC<br>CAA GCT CCT GAA CCG CTC T
Rab6b-F<br>Rab6b-R	GGT TGC CTG GTA GGT GTT GT<br>GCT GCG AAA ATT CAA GTT GG
Gapdh-F<br>Gapdh-R	CCA ATG TGT CCG TCG TGG ATC<br>GTT GAA GTC GCA GGA GAC AAC

Fig. S1

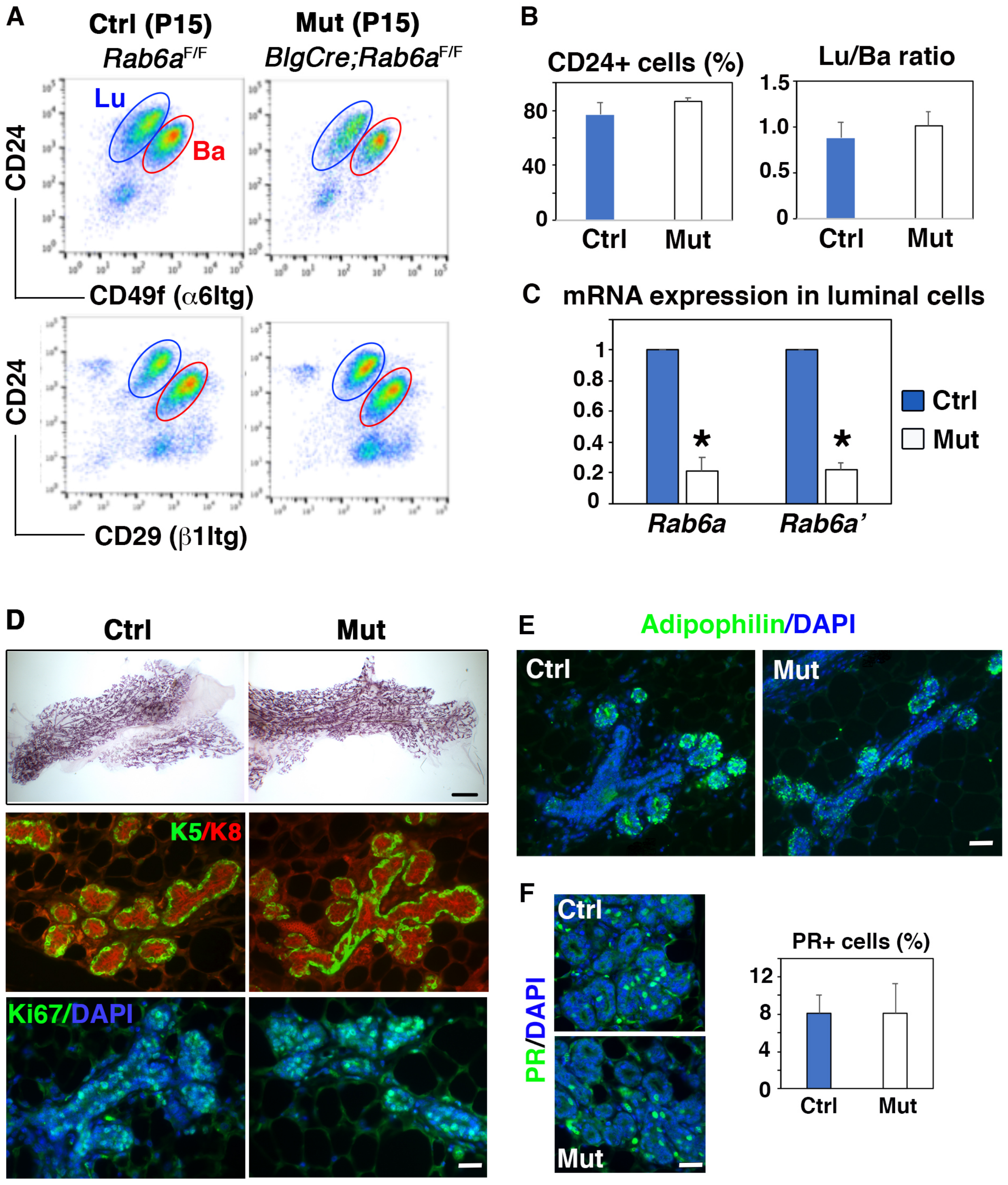

Development • Supplementary information

Figure S1. Mammary phenotype of Blg-Cre; *Rab6a*$^{F/F}$ females at day 15 of pregnancy (P15)

(A) Flow cytometry analysis of mammary cells isolated from control (*Rab6a*$^{F/F}$) and mutant (Blg-Cre; *Rab6a*$^{F/F}$) females at P15. Upper dot-plots: double CD24/CD49f (α6 integrin, α6Itg) immunostaining. Lower dot-plots: double CD24/CD29 (β1 integrin, β1Itg) immunostaining. The gated basal (Ba) and luminal (Lu) cell populations are indicated.

(B) Percentage of CD24+ epithelial cells (left) and ratio between the luminal and basal cell populations (right) calculated from the flow cytometry data obtained at P15. Data are the mean ± SEM of 3 distinct control and mutant cell preparations.

(C) Deletion of *Rab6a* and *Rab6a'* in luminal cells isolated from P15 females, estimated by qPCR. Data are shown as mean ± SEM from 3 distinct mutant and control cell samples. qPCR values were normalized on *Gapdh* and control values were set to 1. *** $p<0.0001$

(D) Whole mount and immunohistological analyses of mammary glands from control and mutant P15 females. Upper panel: carmine staining. Bar, 2mm. Middle and lower panels: double K5/K8 and Ki67/DAPI immunofluorescence labeling. Bar, 30µm.

(E) Immunolocalization of adipophilin in control and mutant mammary tissues at P15. Adipophilin expression is restricted to luminal cells in alveolar buds. Bar, 60µm.

(F) Immunodetection of progesterone receptor (PR) in control and mutant mammary tissues at P15. Left: double PR/DAPI immunofluorescent staining. Bar, 45µm. Right: Percentages of PR+ luminal cells. Data are the mean ± SEM of counting performed on sections through 3 distinct control and mutant mammary glands. About 1500 DAPI-stained nuclei were counted on each section. ns, not significant.

Development • Supplementary information

Fig. S2

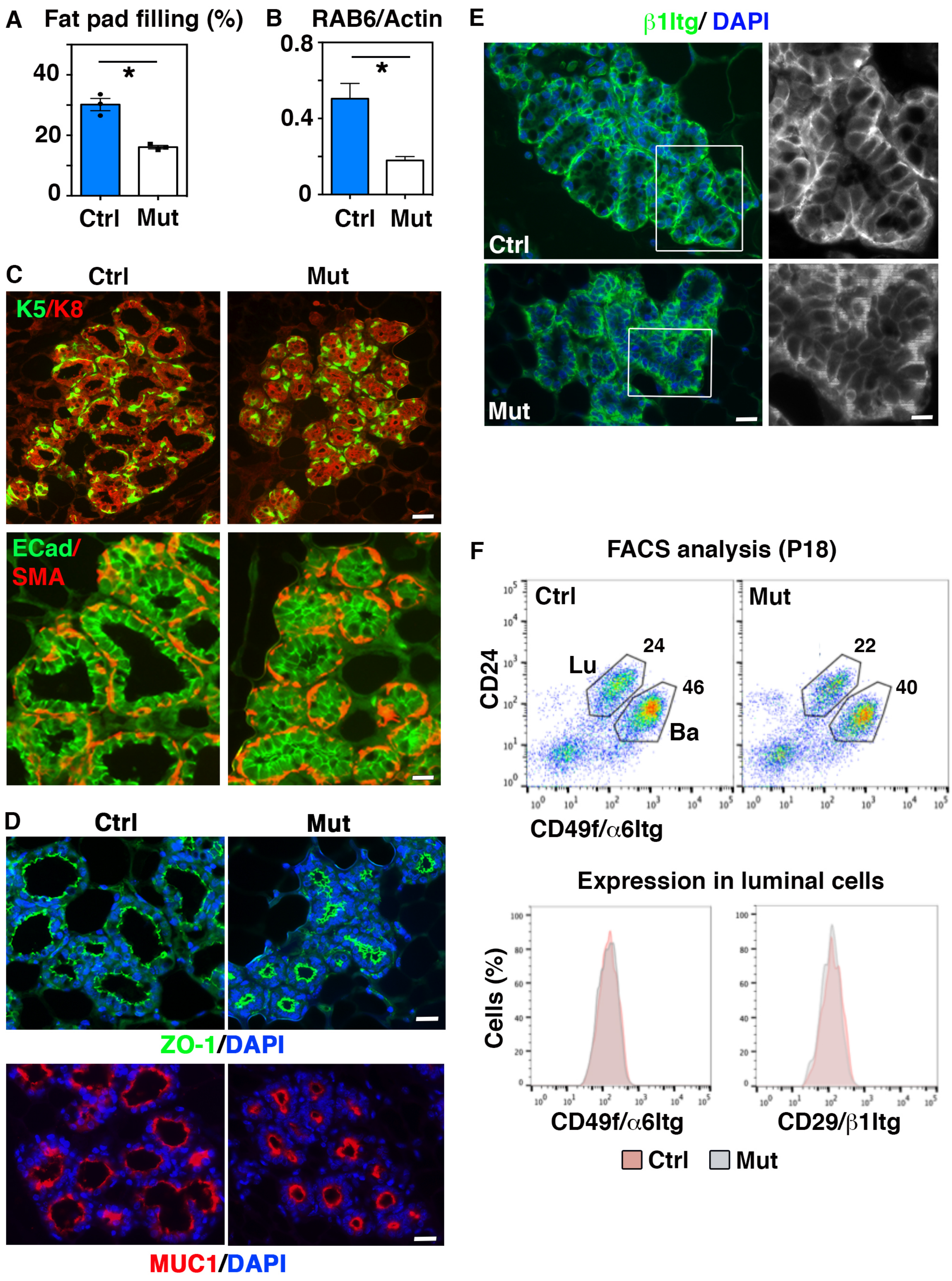

Development • Supplementary information

Figure S2. Mammary phenotype of Blg-Cre; *Rab6a*$^{F/F}$ females at day 18 of pregnancy (P18)

(A) Fat pad filled by control and mutant epithelium at P18. Data are shown as mean ± SEM from three distinct control and mutant females. Each point corresponds to the quantification of two separate tissue sections stained with hematoxylin/ eosin. Representative views of stained sections are shown in Fig. 2B. *p=0.015

(B) Quantification of RAB6 depletion performed on the western blot shown in Fig. 2F. RAB6 expression was normalized to actin. Data are shown as mean ± SEM from four distinct control and mutant mammary gland extracts. *p=0.024

(C) Double immunolabeling for K5/K8 (upper panels; bar, 48µm) and E-cadherin/SMA (lower panels; ECad/SMA; bar, 24µm) in control and mutant mammary epithelium at P18.

(D) Immunolocalization of apical markers in control and mutant mammary tissues at P18. Double labeling of ZO-1/DAPI (upper panels; bar, 25µm) and MUC1/DAPI (lower panels; bar, 35µm).

(E) Immunolocalization of β1 integrin (β1Itg) in control and mutant mammary epithelium at P18. Nuclei are stained with DAPI. Enlarged images of β1Itg expression in the delineated areas are shown on right panels. β1 integrin is strongly expressed by basal myoepithelial cells and is also present at the luminal cell-cell contacts in both mutant and control tissues. Bars, 45µm (left) and 20µm (right).

(F) Flow cytometry analysis of mammary cells isolated from mutant and control mice at P18. Upper panels: Cytograms showing double staining for CD24 and α6Itg (CD49f). The gated basal and luminal cell populations with their respective percentages are indicated. Lower panels: histograms of α6Itg (CD49f, left) and β1Itg (CD29, right) expression in the gated luminal cell population.

Fig. S3

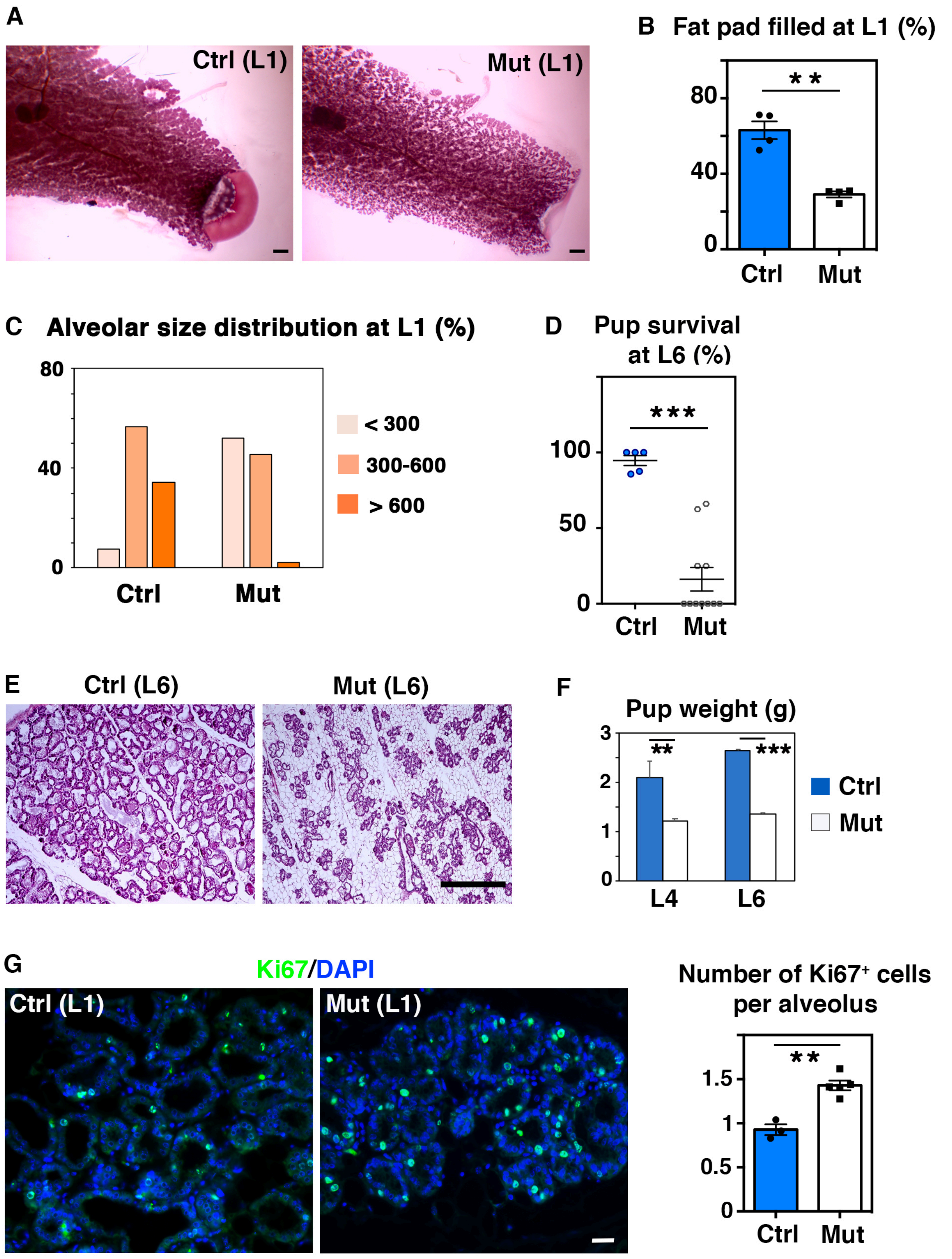

Development • Supplementary information

Figure S3. Mammary phenotype of Blg-Cre; *Rab6a*$^{F/F}$ females at day 1 and 6 of lactation (L1 and L6)

(A) Large views of carmine-stained whole-mounts of mammary glands from control and mutant females at L1. Bar, 1 mm.

(B) Fat pad filled by control and mutant epithelium at L1. Data are shown as mean ± SEM from four distinct control and mutant females. Each point corresponds to the quantification of two separate tissue sections stained with hematoxylin/eosin. Representative views of stained sections are shown in Fig. 3A. **p=0.003

(C) Alveolar size distribution in mutant and control epithelium at L1, estimated in µm. Data are from a pool of measurements performed on two distinct histological sections from 4 control and 4 mutant mammary glands. Pearson's Chi-square test: p<0.001

(D) Survival of pups nursed by control and mutant primiparous dams at L6. Litters from 5 control and 11 mutant females were analyzed. Each point of the graph represents one dam. The percentages reflect the proportion of healthy, viable pups within each individual litter. The dams nursing their entire litter are plotted at 100%, those unable to feed all their pups below 100%. ***p<0.0001

(E) Views of hematoxylin/eosin-stained histological sections through control and mutant mammary glands at L6. Bar, 650µm

(F) Weight of pups nursed by mutant and control dams after 4 and 6 days of lactation. At least 15 pups per control and mutant dams were weighted. Data are shown as mean ± SEM. **p=0.0013, ***p<0.0001

(G) Cell proliferation in control and mutant mammary glands at L1. Left: Double immunofluorescence staining for Ki67 and DAPI in control and mutant mammary epithelium. Bar, 30µm. Right: Quantification of the number of Ki67+ cells per alveolus. Data are shown as mean ± SEM from three control and five mutant females. Each point corresponds to the quantification of five separate tissue sections. **p=0.0018

Development • Supplementary information

Fig. S4

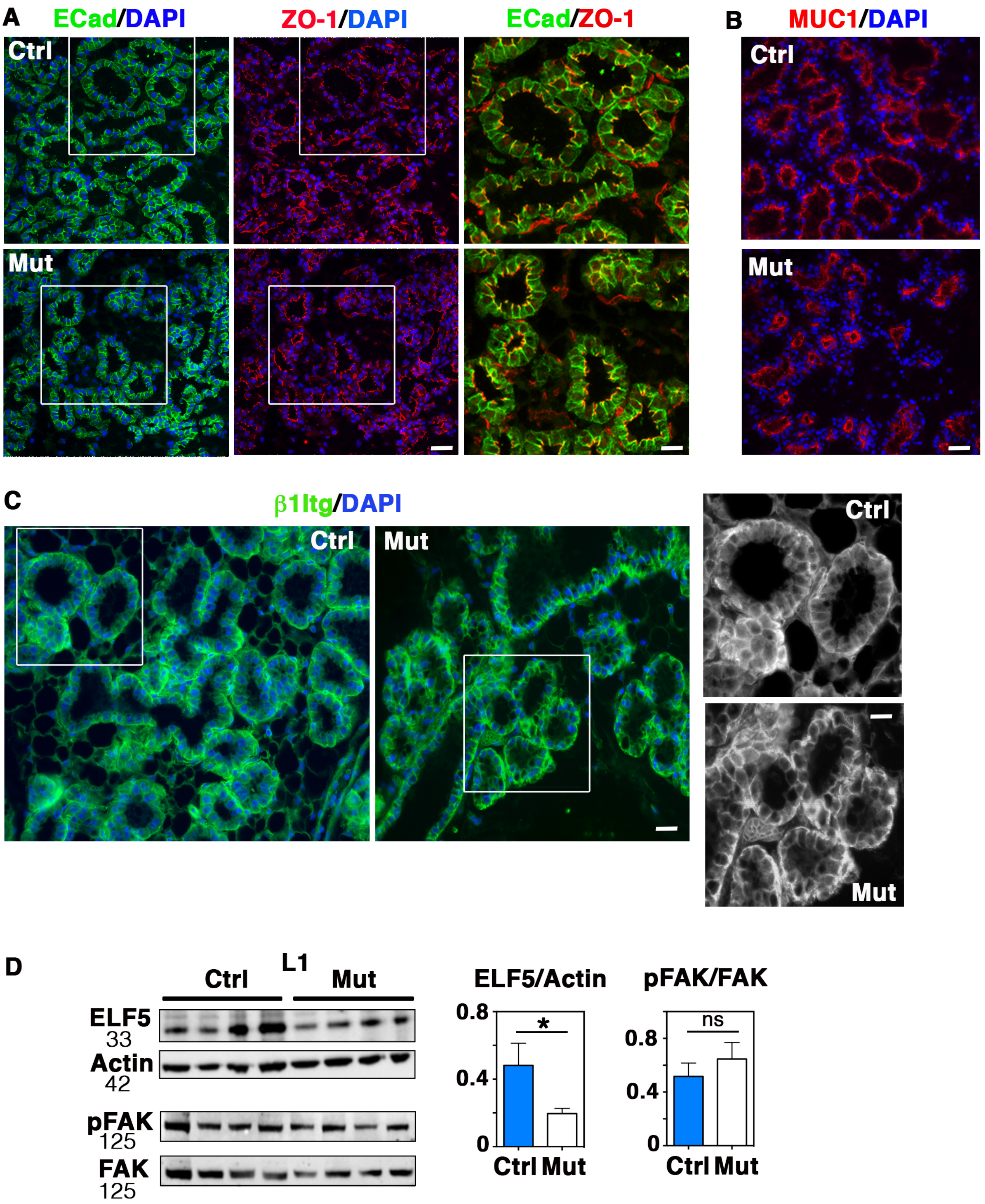

Development • Supplementary information

Figure S4. Mammary phenotype of Blg-Cre; *Rab6a*$^{F/F}$ females at day 1 of lactation (L1)

(A) Double immunofluorescence staining for E-cadherin and ZO-1 in control and mutant mammary epithelium at L1. Enlarged merge images of the delineated areas are shown on right panels. Bars, 48µm (left) and 27µm (right).

(B) Immunodetection of MUC1 in control and mutant mammary epithelium at L1. Nuclei are stained with DAPI. Bar, 48µm

(C) Immunodetection of β1 integrin (β1Itg) in control and mutant mammary epithelium at L1. Nuclei are stained with DAPI. Enlarged images of β1Itg expression in the delineated alveoli are shown on right panels. β1Itg is strongly expressed by basal myoepithelial cells and is also present at the luminal cell-cell contacts in both mutant and control tissues. Bars, 36 µm (left) and 23 µm (right).

(D) Western blots for ELF5, actin, pFAK and total FAK performed on four distinct control and mutant mammary gland protein extracts obtained at L1. MW are indicated in kD. The related quantifications of ELF5/Actin and pFAK/FAK, shown as mean ± SEM, are in the right panels: *p=0.05 (ELF5); ns, not significant

Fig. S5

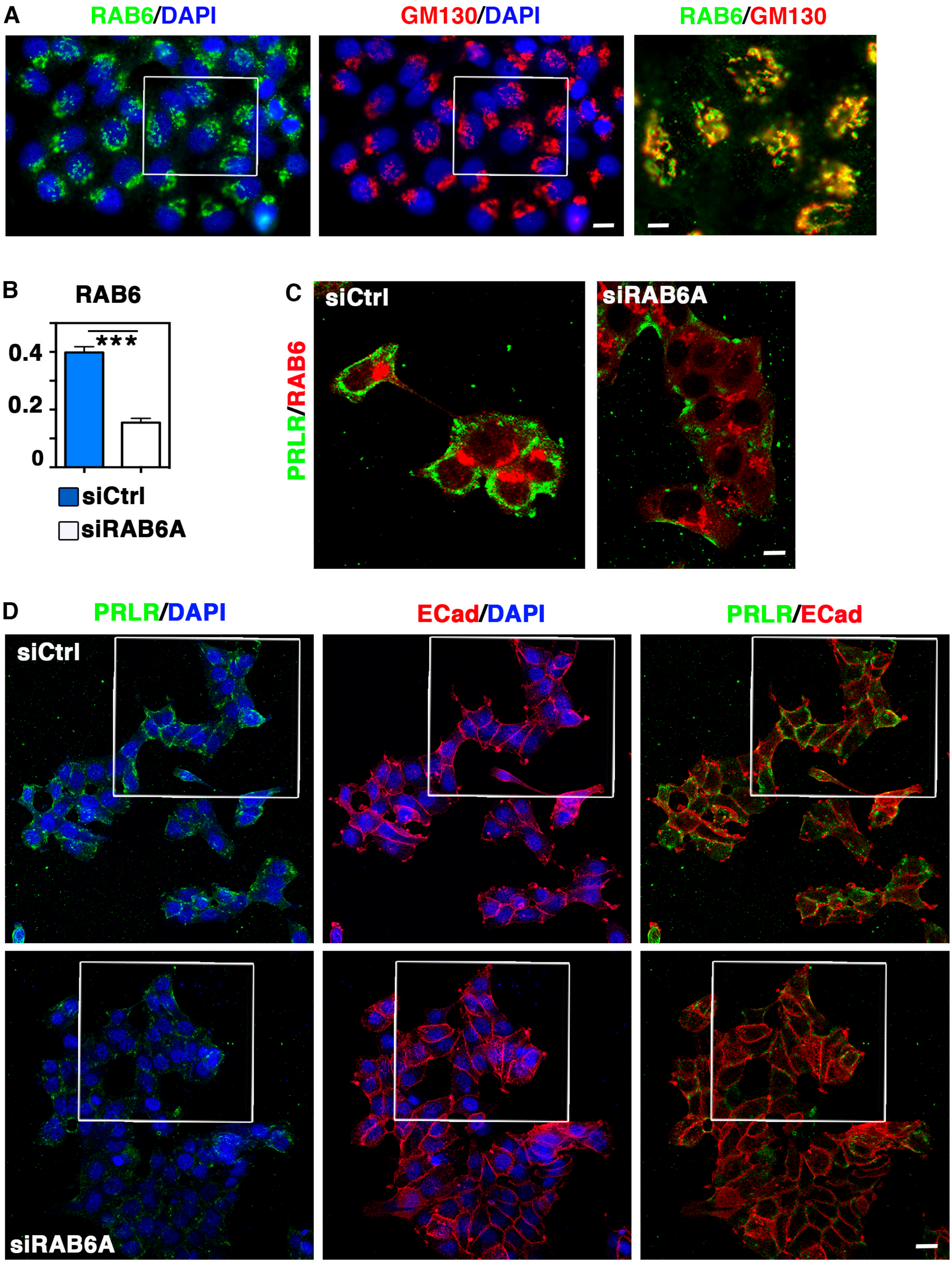

Development • Supplementary information

Figure S5. RAB6A depletion in T47-D cells

(A) Double staining for RAB6 and GM130 in T47-D cells, showing the expected localization of RAB6 in the Golgi. Nuclei are stained with DAPI. Enlarged view of the selected area is shown on the right. Bars, 15µm (left) and 7µm (right).

(B) RAB6 expression in siCtrl and siRAB6A T47-D cells calculated from western blot analyses. An example of western blot is shown in Fig. 6B. Data are the mean ± SEM from 3 distinct siRNA assays with all time points pooled. ***$p < 0.0001$

(C) Immunolocalization of PRLR in unstimulated siCtrl and siRAB6A T47-D cells. Bar, 10µm.

(D) Immunolocalization of PRLR and ECad in unstimulated siCtrl and siRAB6A T47-D cells. Nuclei are stained with DAPI. Images are z-projection of 15 confocal planes across the cells. Enlarged views of the delineated areas are shown in Fig. 6D. Bar, 25µm.
